# Unexpected Thickness-Driven Volume Expansion in Tunicate Cellulose Nanocrystal-Based Multilayer Films

**DOI:** 10.1021/acsmaterialsau.6c00047

**Published:** 2026-08-21

**Authors:** Leïla Garnier, Jean Muller, Pelinsu Demir, Labonno Azad, Pavithra Wijeratne, Christophe Contal, Christine Lancelon-Pin, Michel Tschopp, Yann Battie, Gero Decher, Matthias Pauly, Jean-Luc Putaux, Bruno Jean, Olivier Felix

**Affiliations:** † 69263 Université de Strasbourg, CNRS, Institut Charles Sadron UPR22, Strasbourg F-67000, France; ‡ 55139 Univ. Grenoble Alpes, CNRS, CERMAV, Grenoble F-38000, France; § Université de Lorraine, LCP-A2MC, Metz F-57000, France; ∥ International Center for Frontier Research in Chemistry, Strasbourg F-67083, France

**Keywords:** layer-by-layer assembly, nanocomposite thin films, nanocellulose, tunicate cellulose nanocrystals, unusual thickness jump, porous mikado-like structure, ellipsometry

## Abstract

Cellulose nanocrystals are rod-like nanoparticles obtained from different cellulose sources (plants, bacteria, algae, or tunicates) and represent a class of renewable, biodegradable, and mechanically robust nanomaterials with great potential for the design of advanced functional materials. In this study, we report on the preparation of multilayer composites based on tunicate cellulose nanocrystals and poly­(vinyl amine) using the layer-by-layer assembly technique. The assembly of these nanostructured films was characterized by ellipsometry. Surprisingly, films composed of tunicin nanocrystals and poly­(vinyl amine) showed a highly unusual film growth behavior by dipping, spray- and spin-assisted assembly, and grazing incidence spraying methods, which contrasted with previous reports. When the film thickness reached approximately 50–60 nm, the deposition of additional layers induced a sharp increase in thickness that nearly doubled the volume of the film by expansion in the direction normal to the surface. This behavior was also observed by atomic force microscopy, ruling out any optical artifact due to ellipsometry. Ellipsometry measurements revealed that this thickness jump (i) was independent of the assembly conditions, (ii) was associated with an increase in film porosity, and (iii) clearly correlated with the aspect ratio and rigidity of the tunicate cellulose nanocrystals. This unexpected phenomenon was not observed for films assembled from either shorter cellulose nanocrystals extracted from cotton or wood, or from longer and more flexible cellulose nanofibrils in combination with poly­(vinyl amine). Such a pronounced structural transition has never been previously observed, and its origin remains unexplained despite the experiments performed to date.

## Graphical abstract

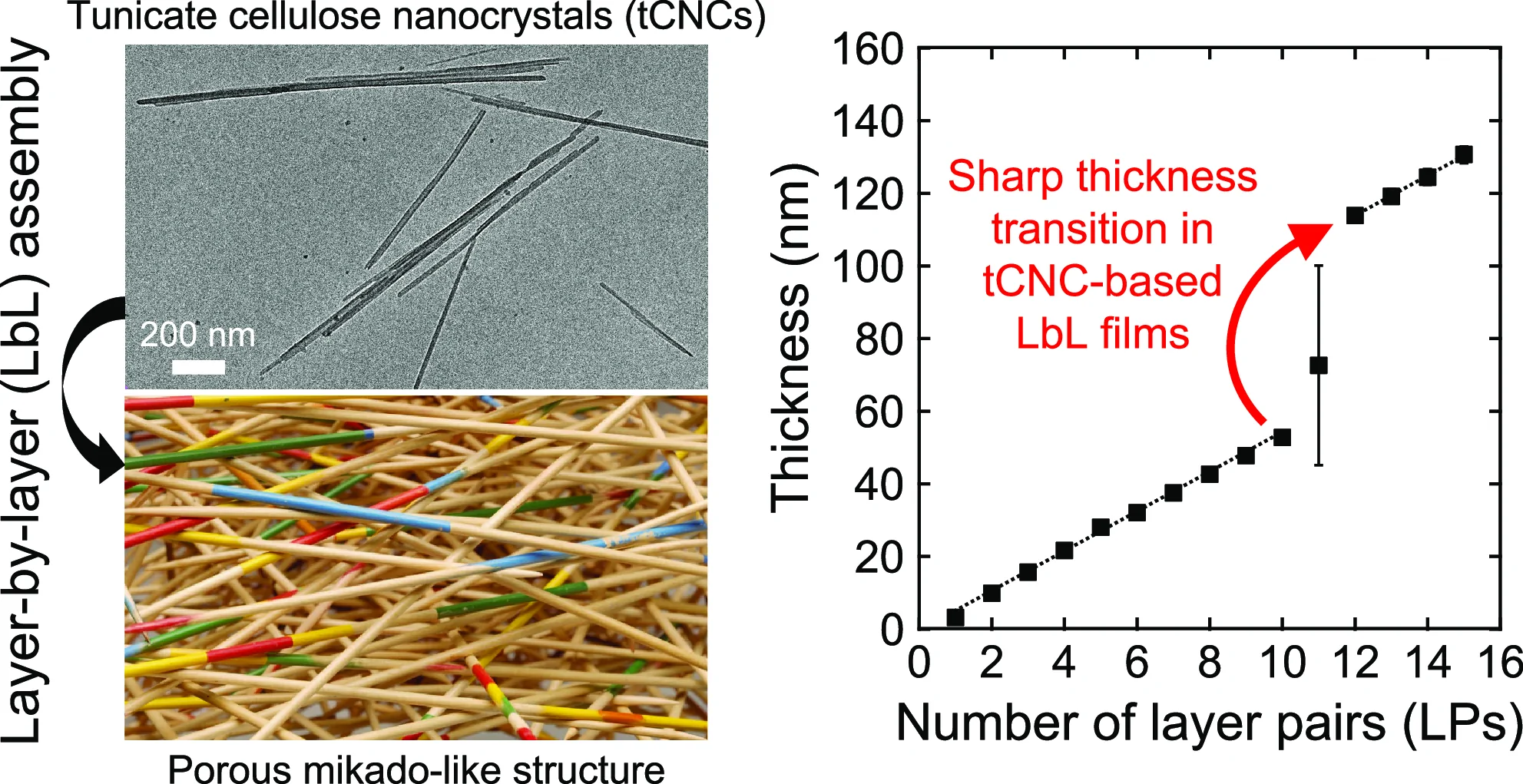

## 1 Introduction

The transition toward biosourced materials as sustainable alternatives to petroleum-based products requires the use of bioderived building blocks that meet several key criteria.^1^ These include large-scale industrial availability from abundant natural resources, noncompetition with food supply chains, and the ability to exhibit tunable structural organization. Nanocellulose, encompassing both cellulose nanofibrils (CNFs) and cellulose nanocrystals (CNCs), meets most of these requirements.^2^ Derived from an almost inexhaustible biomass source, nanocellulose now benefits from industrial-scale production capabilities, supported by the expanding utilization of lignocellulosic feedstocks. CNCs are generally short, rigid, and highly crystalline rod-like particles typically produced by strong acid hydrolysis, whereas CNFs are long, and flexible fibrils containing both crystalline and desorganized regions, usually obtained by chemical modification and mechanical fibrillation. Both CNCs and CNFs combine advantageous physicochemical properties such as low density, high tensile strength, adjustable aspect ratio and morphology, and high specific surface area.^2,3^ Additionally, their versatile surface chemistry enables a broad range of chemical modifications, making nanocellulose an attractive candidate for the design of advanced functional nanostructured materials.

Within this context, the layer-by-layer (LbL) assembly technique^4^ has attracted considerable attention for the fabrication of nanocellulose/polymer thin films and coatings with enhanced functionalities.^5–8^ LbL assembly is recognized for its simplicity, versatility, environmental compatibility, and cost-effectiveness. It enables the fabrication of multilayered films with precisely controllable nanoscale architecture, incorporating a wide range of constituents, including polyelectrolytes, small organic and inorganic molecules, biomacromolecules, and colloidal materials, on different solvent-accessible surfaces, regardless of their shape or composition.^9–11^ The film formation and structural integrity in LbL-assembled systems are governed by diverse intermolecular interactions, such as electrostatic interactions, hydrogen bonding, hydrophobic forces, and covalent bonding. A key advantage of this technique for the preparation of nanocomposites lies in the ability to tailor the structure through the deposition sequence, which determines the layer sequence, and the deposition conditions, which control, for example, the adsorbed amount per layer.

The first report on nanocellulose/polymer multilayer films was published by Podsiadlo et al. in 2005,^12^ marking a key milestone in the integration of nanocellulose into LbL architectures. Since then, the intersection of nanocellulose and LbL assembly has evolved into an active research domain.^5–8^ Both CNFs and CNCs have been successfully incorporated in conjunction with various polymers in LbL films to develop advanced functional materials for applications in separation membranes, flexible electronics, bioenergy devices, packaging and advanced optical coatings.^6,7^

Recently, we demonstrated that LbL assembly can be effectively combined with grazing incidence spraying (GIS)^13^ to fabricate helicoidal CNF-based multilayer films, in which the orientation of CNFs can be controlled independently in each layer.^14^ Before extending this approach to less flexible nanoparticles such as CNCs, we investigated the growth behavior of CNC-based multilayer films prepared using different LbL deposition methods. tCNCs prepared from tunicin, the cellulose from the sea animals tunicates, were selected as building blocks due to their high aspect ratio and exceptional mechanical stiffness,^15,16^ characteristics that make them particularly suitable for aligning and constructing complex LbL architectures. The first fabrication of thin multilayer films composed of tCNCs by LbL assembly was reported by Podsiadlo et al. in 2007.^17^ They demonstrated that the resulting films exhibited a linear thickness growth and displayed strong antireflective properties, attributed to the high aspect ratio of the tCNCs, which promotes the formation of a “porous matchsticks pile” structure (Figure 1). More recently, Moon et al. confirmed the regular growth behavior, as well as the porous structure and optical properties of tCNC-based films.^18^ As for CNFs,^8^ it was already observed that the structure and conformation of the polymer (neutral or charged) have an influence on the growth and structure of CNC-based LbL films.^6^

**Figure 1:**
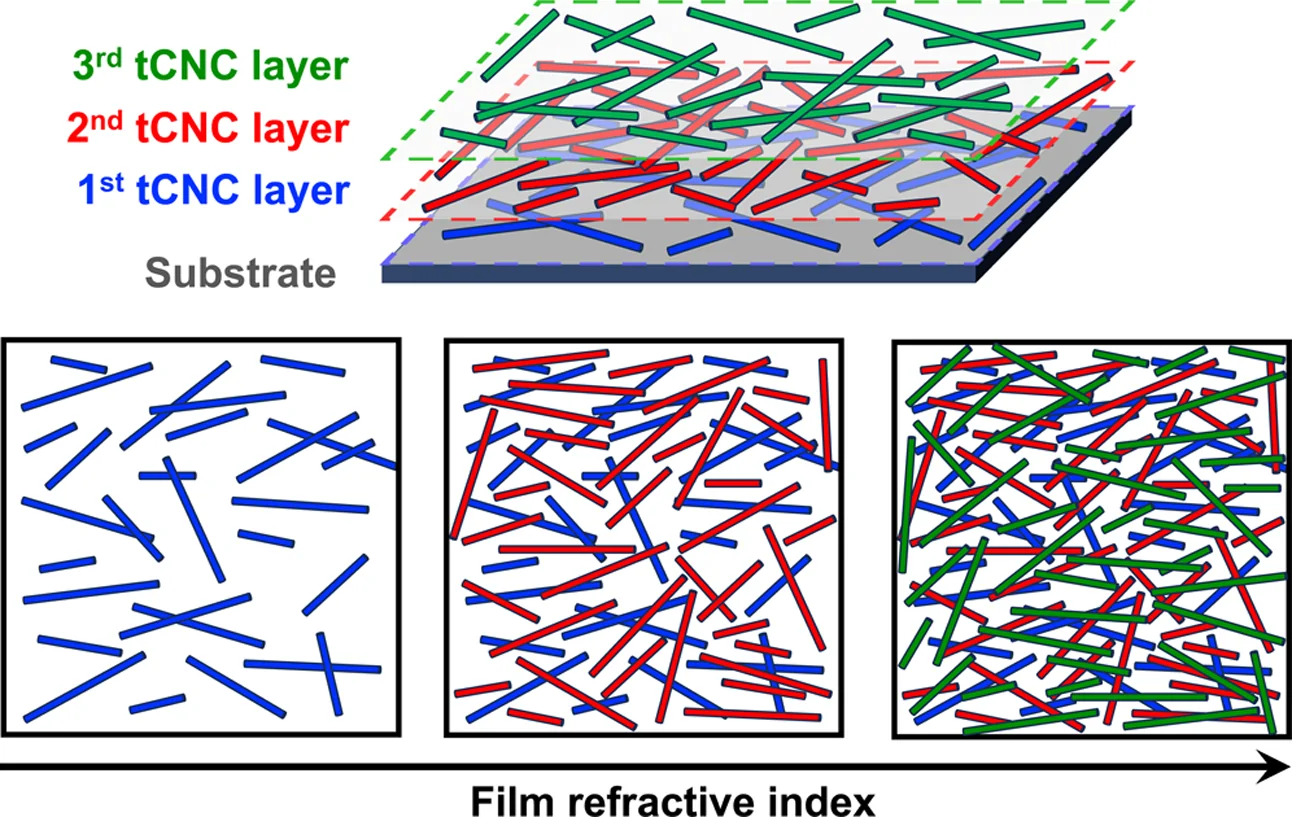
(top) Schematic representation of the LbL principle for the deposition of the first three tCNC layers and (bottom) top-view projection of the structural model of the first three layers of a tCNC-based film illustrating how the film porosity and refractive index evolve through the deposition of additional nanocellulose layers (adapted from refs 17 and 18 ^#^).^#^Adapted from Carbohydrate Polymers, 282, Moon, S. M.; Kim, D.-W.; Lee, S.; Eom, T.; Jeon, S. H.; Shim, B. S., Precisely Tuned Photonic Properties of Crystalline Nanocellulose Biocomposite Coatings by Gradually Tailored Nanoarchitectures, 119053, copyright (2022), with permission from Elsevier.

In this study, we report on an unusual thickness growth behavior observed during the fabrication of multilayer films composed of tCNCs and PVAm, assembled for the first time by LbL assembly and GIS. To investigate this phenomenon, films were fabricated using dipping, spray-assisted, spin-assisted, and GIS methods, and their thickness growth was monitored by ellipsometry. Independent of the deposition method, films exhibited a sharp and pronounced increase in thickness at approximately 50–60 nm, corresponding to a near doubling of the film volume in the direction normal to the substrate, a behavior not previously reported in the literature. This variation was associated with a drastic decrease in the tCNC volume fraction, resulting in a rapid increase in the porous volume. The porous mikado-like structure of these films was characterized by scanning electron microscopy (SEM) and atomic force microscopy (AFM). The occurrence of this structural transition during film buildup was further confirmed by AFM step-profile measurements, ruling out potential optical artifacts associated with ellipsometry. Ellipsometry, SEM, and AFM analyses were subsequently used to assess the influence of the nanocellulose and polymer nature, as well as the deposition methods and conditions on this unusual thickness jump and to explore its possible origin.

## 2 Materials and Methods

### 2.1 Materials

Poly­(sodium 4-styrenesulfonate) (PSS, *M*_*W*_ = 70,000 g·mol^–1^), poly­(allylamine hydrochloride) (PAH, *M*_*W*_ = 50,000 g·mol^–1^), poly­(dimethyldiallylammonium chloride) (PDDA, 20 wt % in H_2_O, *M*_*W*_ = 100,000 – 200,000 g·mol^–1^) and poly­(vinyl alcohol) (PVA, Mowiol 10–98, *M*_*w*_ ∼ 61,000 g·mol^–1^) were purchased from Sigma-Aldrich (Lyon, France). Poly­(ethyleneimine) (PEI, trade name Lupasol WF, *M*_*W*_ ∼ 25,000 g·mol^–1^) and poly­(vinylamine) (PVAm, trade name Lupamin 9095, 20 wt % in H_2_O, *M*_*W*_ = 340,000 g·mol^–1^) were freely provided by BASF (Ludwigshafen, Germany). Tunicate and cotton CNC (tCNC_CV_ and cCNC, respectively) suspensions (0.5 and 3 wt % in H_2_O, respectively) were prepared at CERMAV (Grenoble, France) from the mantles of tunicates (*Halocynthia roretzi*) and cotton linters from Buckeye Cellulose Corporation, respectively, using protocols described elsewhere.^19^ Additionally, a xyloglucan (XG) solution was also prepared at CERMAV using XG from tamarind seeds (grade 3S) provided by Dainippon Pharmaceutical Co. and solubilized according to a previously reported protocol.^20^ Commercial tunicate and wood CNC (tCNC_Lab_ and wCNC) suspensions (0.5 and 6 wt % in H_2_O, respectively) were purchased from Cellulose Lab (Fredericton, Canada). All CNCs used in this study were prepared by sulfuric acid hydrolysis, meaning that they bear sulfate half-ester groups with a low p*K*_a_ (typically around 1–2).^21^ These groups are essentially fully deprotonated at neutral and basic pH, leading to an almost constant negative surface charge. The sulfur content of tCNC_CV_ and tCNC_Lab_ was 0.31 ± 0.04% (0.097 mmol·g^–1^) and 0.92 ± 0.04% (0.287 mmol·g^–1^), respectively, as determined by inductively coupled plasma optical emission spectrometry (ICP-OES). These values can be translated into a surface charge density by taking into account the dimensions of the nanoparticles. Approximate values of 0.4 and 0.8 e-·nm^–2^ were obtained for tCNC_CV_ and tCNC_Lab_, respectively. The carboxymethylated CNFs (wCNFs), bearing carboxylic groups with a p*K*_a_ value around 4–5,^8,22^ were supplied by Cellulose Lab as a powder. Milli-Q water (resistivity of 18.2 MΩ·cm) used for the preparation of all solutions, suspensions, and experiments was produced using a Milli-Q Advantage A10 water purification system (Merck-Millipore, Molsheim, France). All chemicals were used as received without further purification.

PVAm, PAH and PDDA were dissolved in Milli-Q water to obtain solutions at a concentration of 1 g·L^–1^ with pH values around 8–9 for PVAm and 4–5 for PAH and PDDA. These polycation solutions showed positive zeta potential values (>25 mV). In these solutions, PVAm, PAH and PDDA had a hydrodynamic radius around 4, 5, and 0.5 nm, respectively, measured by dynamic light scattering. For the fabrication of (PSS/PAH)_n_ films, PAH and PSS were dissolved in Milli-Q water containing 0.5 M NaCl to prepare solutions at concentrations of 0.6 and 0.3 g·L^–1^, respectively. PVA was diluted in Milli-Q water to obtain a solution at a concentration of 5 g·L^–1^ with a pH around 7. When necessary, the pH of the PVAm solution was adjusted using a 0.5 M solution of either NaOH or HCl. PEI was dissolved in Milli-Q water to a final concentration of 2.5 g·L^–1^, exhibiting a pH value around 9–10. Its hydrodynamic radius was around 1 nm.

CNC suspensions were used as received and further diluted to the desired concentration using Milli-Q water. tCNC_CV_ and cCNC suspensions at concentrations of 0.5 and 3 wt %, respectively, were diluted to prepare suspensions at 0.04 wt % (0.4 g·L^–1^), unless otherwise specified. The suspensions were stirred at room temperature for at least 1 day before use. The same protocol was used to prepare suspensions at 0.4 g·L^–1^ (unless otherwise specified) of tCNC_Lab_ and wCNC with an initial concentration of 0.5 and 6 wt %, respectively. All CNC suspensions showed an acidic pH (around 4–5) and high negative zeta potential values (below −40 mV; especially −45.3 ± 7.1 mV for tCNC_CV_ and −56.8 ± 5.4 mV for tCNC_Lab_). wCNFs were initially dispersed in Milli-Q water at a concentration of 0.5 g·L^–1^ and then filtered through cotton wool to remove the largest particles. The concentration of the resulting suspension was determined to be approximately 0.4 g·L^–1^ based on dry mass measurements. The pH of the wCNF suspension in Milli-Q water was measured to be around 7–8, meaning that CNFs are negatively charged at neutral and basic pH. It showed a high negative zeta potential value (below −50 mV). Xyloglucan was solubilized in Milli-Q water to prepare solutions at 0.04 wt % (0.4 g·L^–1^) under stirring and heating at 50 °C. XG solution exhibited a pH value around 7. These nanocellulose suspensions remained stable for several weeks/months under refrigerated storage conditions.

### 2.2 Substrates

Silicon wafers (200 mm in diameter, (100) orientation) used as substrates for the fabrication of LbL films were purchased from WaferNet, Inc. (San Jose, CA, USA). Before use, wafers were cut, immersed in a 0.1 M NaOH solution for 15 min, thoroughly rinsed with Milli-Q water, immersed in a 50:50 (v/v) H_2_O:ethanol solution, and then sonicated for 15 min in an ultrasound bath. After drying under compressed air, substrates were cleaned/activated by plasma treatment for 3 min at high RF power (∼30 W) in a PDC-002 plasma cleaner (Harrick Plasma, USA).

### 2.3 LbL Deposition

LbL films were fabricated using dipping, orthogonal spray-assisted assembly, spin-assisted assembly, or grazing incidence spraying. All films were assembled on substrates precoated with a PEI precursor layer deposited by dipping (15 min followed by three rinsing steps of 2 min each in Milli-Q water and drying under compressed air).

For LbL films prepared by dipping, the PEI-modified substrate was dipped in the CNC suspension for 5 min, followed by three rinsing steps of 2 min each in Milli-Q water. The substrate was then dried under compressed air. The same procedure was used for the deposition of PVAm, PEI, PAH, and PDDA. One complete deposition of the two alternating components constituted a single layer pair (LP), and the cycle was repeated until the desired film thickness was achieved. The fabrication of (PSS/PAH)_n_ films was performed using the procedure described previously.^23^

The orthogonal spraying-assisted deposition of CNCs and polycations was carried out using hand-operated air pump spray cans made of poly­(ethylene) and poly­(propylene). Each can was filled with the corresponding suspension/solution and pressurized by manual pumping. Spraying was carried out perpendicularly to the receiving surface, which was positioned vertically. Each solution was sprayed onto the surface for 5 s, followed by a rinsing step with Milli-Q water for 10 s. Before ellipsometric measurements, the films were dried under compressed air.

For spin-assisted LbL assembly, the substrate was mounted on a spin coater (Laurell Technologies, North Wales, PA, USA). The substrate was rotated at 8000 rpm, and approximately 0.5 mL of CNC suspension was dispensed dropwise onto the center of the rotating substrate. The latter was spun until dry, rinsed with 1 mL of Milli-Q water, and spun again until completely dry. The same procedure was repeated for the deposition of the PVAm layer. One complete deposition of CNCs and PVAm constituted a single LP, and the cycle was repeated until the desired film thickness was reached.

For GIS deposition, an aqueous CNC suspension was sprayed onto a PEI-coated silicon wafer for 60 s using a custom-built spraying system. The setup comprises gas-flow controllers (model Red-Y, Voegtlin, Aesch, Switzerland), liquid-handling pumps (model M50, VICI, Schenkon, Switzerland), and a two-fluid spray nozzle (internal diameter: 300 μm, Spraying Systems, Wheaton, IL, USA). The nozzle was positioned 1 cm from the substrate. The angle between the spray cone main axis and the receiving substrate was approximately 13°. The liquid flow rate was set to 1 mL·min^–1^, and the air flow to 40 L·min^–1^. PVAm was deposited by orthogonal spraying for 5 s using a hand-operated air pump spray can. Each deposition of CNC and PVAm was followed by a rinsing step with Milli-Q water for 10 s using an air pump spray can. Finally, the substrate was dried under compressed air flow.

### 2.4 Ellipsometry

Film thickness measurements were performed using a PLASMOS SD 2300 ellipsometer operating at a wavelength of 632.8 nm and an incidence angle of 70°. We used a single-layer optical model with a fixed refractive index of 1.465 for data fitting, corresponding to an average between the refractive indices of cellulose (≈1.46 for pure CNC films,^24^ *n*_*CNC*_ ≈ 1.55 ^25^), PVAm (typically *n* = 1.3–1.7 for polymers), and air (*n* = 1). Each measured thickness value was the average of 10 measurements taken at different positions on the sample. This procedure allowed a rapid determination of the thickness and sufficient accuracy for comparing the buildup and homogeneity of the films investigated in this study.

Conventional spectroscopic ellipsometry and Mueller matrix ellipsometry measurements were performed at an angle of incidence of 70° using a photoelastic modulator–based ellipsometer (Horiba-UVISEL). Only the first three columns of the Mueller matrix were measured.

### 2.5 Atomic Force Microscopy

Tapping-mode AFM was performed using a Multimode 8-HR AFM instrument (Bruker Nano Surface, CA, USA) equipped with a Nanoscope V controller. Aluminum-coated silicon cantilevers model RTESPA-300 from Bruker, with a typical resonance frequency of 300 kHz, a nominal spring constant of 40 N·m^–1^, and a nominal tip radius below 10 nm were used. Height and phase images were acquired simultaneously at a constant scan rate of 1 Hz with a resolution of 512 × 512 pixels. Image processing was performed using the NanoScope Analysis software, version 1.9 (Bruker Nano Surface, CA, USA). Prior to analysis, all images were flattened using a polynomial correction, mainly a second-order polynomial and a plane fit filter for tilt correction. For scratch imaging, the height profile was extracted from the average of the entire image, perpendicular to the scratch on the film. The film thickness was determined from the relative height difference between two distinct regions on the sample surface: the top of the film and the exposed substrate.

### 2.6 Scanning Electron Microscopy

For SEM observations, the surface of (tCNC/PVAm)_n_ films was coated with a 4 nm-thick conductive layer of Au/Pd under vacuum in a Safematic sputter coater (Eden Instruments). Samples were observed in secondary electron mode with an FEI Quanta-FEG 250 (Thermo Fisher Scientific, USA) scanning electron microscope equipped with a field-emission gun and operating at an accelerating voltage of 2.5 kV.

### 2.7 Transmission Electron Microscopy (TEM)

Droplets of dilute aqueous suspensions (ca. 0.001 wt %) were deposited onto carbon-coated TEM copper grids previously glow-discharged in a PELCO easiGlow station to make the carbon surface hydrophilic. After a few minutes, the excess liquid was blotted with filter paper. Some preparations were left to air-dry, while others were negatively stained with 2% uranyl acetate before drying. Images of both types of specimens were recorded under low-dose illumination with a JEOL JEM-2100-Plus microscope operating at 200 kV and equipped with a Gatan Rio 16 digital camera.

### 2.8 Dynamic Light Scattering and Zeta Potential

Hydrodynamic size of the polyelectrolytes in solution was determined with a Zetasizer Nano ZS instrument (Malvern Panalytical, Malvern, UK). The measurement was carried out after a 3 min equilibration period at 25 °C. For each sample, the reported hydrodynamic radius corresponds to an average value of five independent measurements. Zeta potential measurements were performed using a Zetasizer Nano ZS instrument (Malvern Panalytical, Malvern, UK) equipped with a specific cuvette containing electrodes to allow the passage of the electric current through the suspension. For each sample, the reported zeta potential corresponds to an average value of three independent measurements, each consisting of at least 14 runs. All zeta potential values are given with a precision of ± 0.1 mV, corresponding to the instrument’s measurement accuracy.

### 2.9 Inductively Coupled Plasma Optical Emission Spectrometry

The sulfur content of tCNC_CV_ and tCNC_Lab_ samples was measured with a Varian 720 spectrometer equipped with a quartz Meinhard nebulizer and a cyclone spray chamber.

## 3 Results and Discussion

### 3.1 Assembly of tCNC-Based Multilayers by Dipping and Validation of the Unusual Thickness Transition

In this work, two sources of tCNCs, a commercial one supplied by Cellulose Lab (tCNC_Lab_) and one prepared at CERMAV (tCNC_CV_), were used to fabricate multilayer films composed of tCNCs and PVAm via LbL assembly. Prior to film assembly, the morphology of tCNC_Lab_ and tCNC_CV_ was characterized by TEM from dilute suspensions dried on a carbon film (Figure 2, Figure S1) and by AFM from a submonolayer (dispersion prepared by dipping the substrate in a dilute suspension) deposited on a PEI layer (Figure S2). The two types of tCNCs are markedly different: while tCNCs_Lab_ are long whisker-like particles likely corresponding to single cellulose crystallites (Figure 2a), tCNC_CV_ are shorter, spindle-like bundles of a few crystallites (Figure 2b). This difference likely results from the respective methods of extraction and purification of the CNCs from tunicate tissues. The dimensions and size distributions of the particles determined from AFM images are summarized in Supporting Information Figure S2 and Table S1. The corresponding aspect ratio was approximately 90 for tCNC_Lab_ and 50 for tCNC_CV_. These values were in good agreement with those reported by the supplier (Cellulose Lab, aspect ratio around 75) and previously published work (tCNC aspect ratio between 50 and 100).^19,26^ It should be noted that determining the dimensions of nanocelluloses is challenging due to their diverse morphologies, high variability in sample preparation, and the limitations of imaging techniques.^27,28^

**Figure 2:**
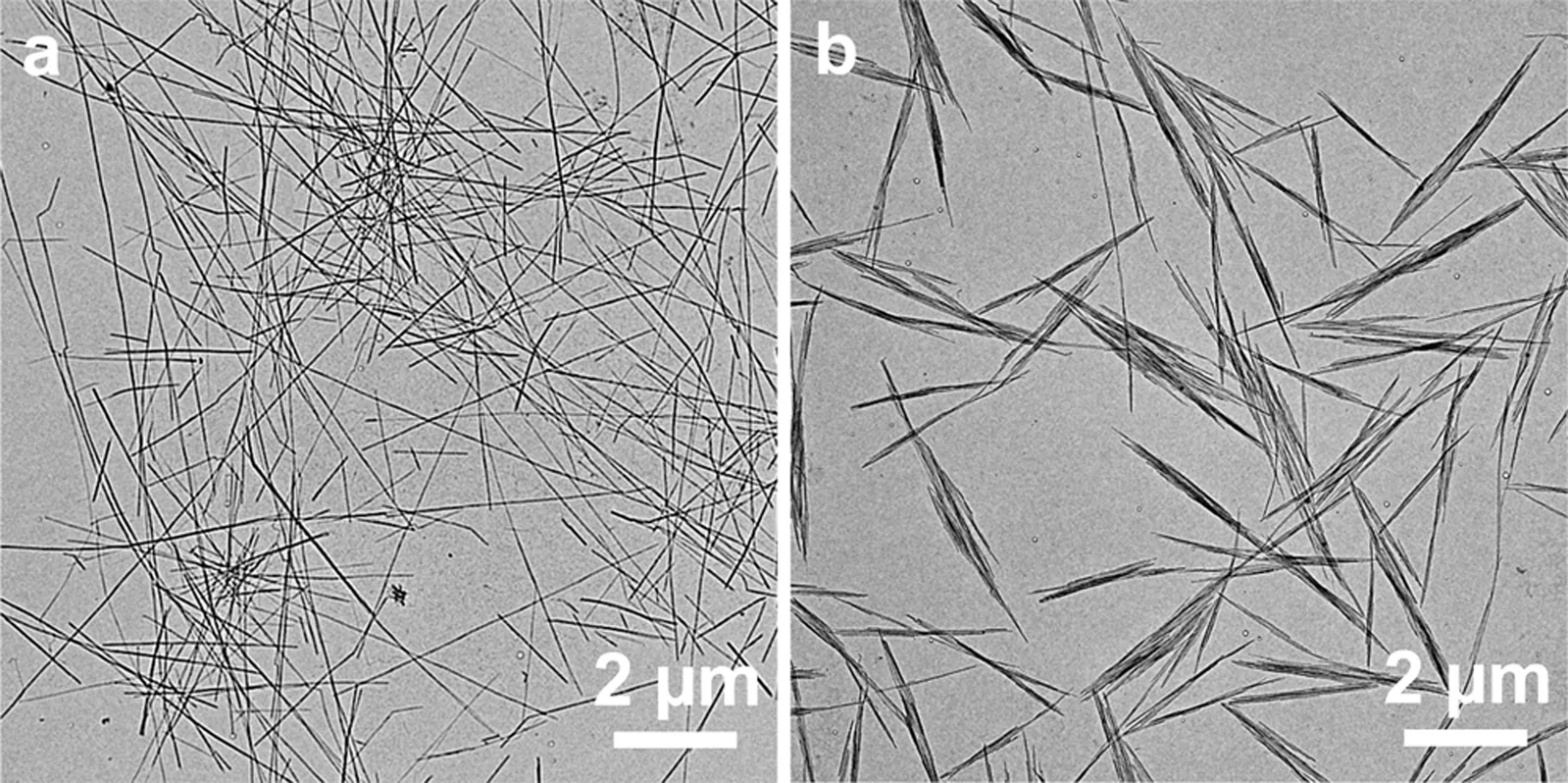
TEM images of preparations from tCNC_Lab_ (a) and tCNC_CV_ (b) suspensions.

tCNC_Lab_ and tCNC_CV_ were then used to fabricate multilayer films composed of tCNCs and PVAm via LbL assembly and GIS. Similarly to polyelectrolyte complexation in solution,^29^ the formation of the tCNC-based multilayers is entropically driven by the release of the counterions during the complexation between the negatively charged tCNC rods and the positively charged PVAm.^30^ The counterions associated with each polyelectrolyte group are expelled allowing the formation of ionic bonds between the oppositely charged moieties. Indeed, during LbL-assembly, the film is in a neutral state due to intrinsic (polyelectrolyte/polyelectrolyte interactions) and extrinsic (polyelectrolyte/counterion complexes) compensation.^31^ The preparation of (tCNC/PVAm)_n_ films was first conducted by conventional dipping using a tCNC suspension and a PVAm solution, both prepared in ultrapure water at a concentration of 0.4 and 1 g·L^–1^, respectively. A dipping time of 5 min was selected as it enabled efficient film growth while minimizing processing time. Longer dipping times (10 or 60 min) resulted in a similar thickness evolution (Figure S3). The pH of the tCNCs, PVAm, and rinsing solutions was not adjusted before film assembly on silicon wafers, unless otherwise specified. The LbL assembly process implied that the film was exposed either to an acidic tCNC suspension (pH around 4–5) when tCNCs were deposited on a film terminated by a PVAm layer or to a basic PVAm solution (pH around 8–9) when PVAm was deposited on a film terminated by a tCNC layer. In view of the p*K*_a_ of the ionizable groups and pH values of the corresponding suspensions/solutions, such pH variations led certainly to changes in the effective charge of tCNC and PVAm but in the direction of a higher degree of ionization. The intermediate rinsing steps with Milli-Q water brought back the pH of the medium to a neutral pH. As all deposition steps during the film buildup were carried out under identical conditions, a similar charge variation should take place during the alternate deposition of nanocellulose and polyelectrolyte.

The buildup of (tCNC/PVAm)_n_ films was monitored by monochromatic ellipsometry (Figure 3). Figure 3a shows the evolution of the film thickness with the increasing number of (tCNC/PVAm) deposition cycles. In contrast to previous reports,^17,18^ where tCNC-based multilayers assembled with a polyelectrolyte display a regular growth, both types of tCNC-based films in this study exhibited a highly unusual thickness growth behavior. These films grew linearly up to a critical thickness of approximately 50–60 nm, beyond which an unexpected thickness jump occurred, nearly doubling the total film thickness.

**Figure 3:**
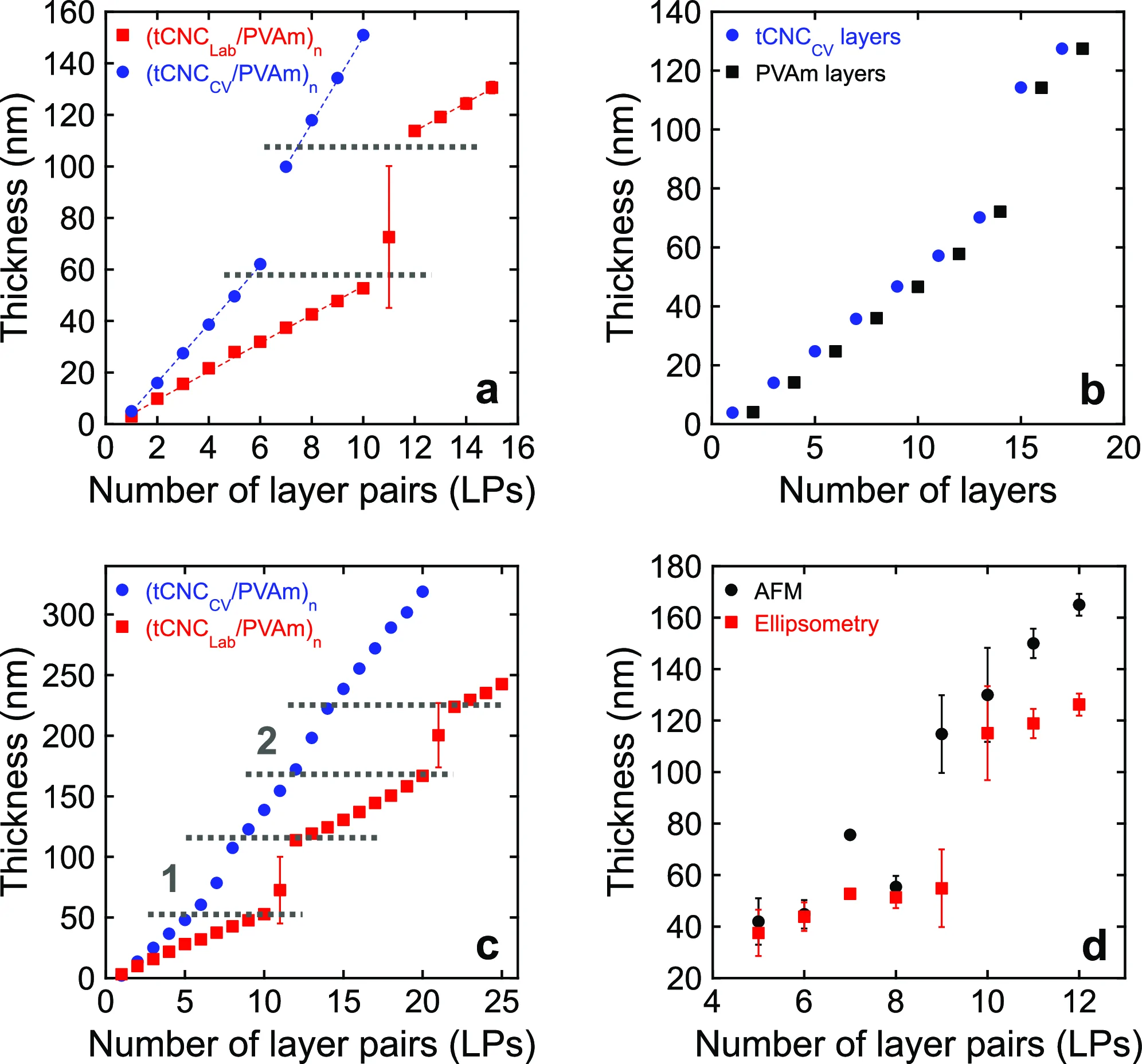
(a) Variation of the thickness of (tCNC_Lab_/PVAm)_n_ (red) and (tCNC_CV_/PVAm)_n_ (blue) films prepared by dipping as a function of the number of layer pairs monitored by ellipsometry after each deposited layer pair. The dotted lines outline the area where the thickness jump occurs. b) Variation of the thickness of (tCNC_CV_/PVAm)_n_ films prepared by dipping measured by ellipsometry after the deposition of each layer (tCNC_CV_ in blue, PVAm in black). c) Multiple thickness jumps observed for (tCNC_Lab_/PVAm)_n_ (red) and (tCNC_CV_/PVAm)_n_ (blue) films during the growth measured by ellipsometry. (d) Comparison of the thickness values for (tCNC_Lab_/PVAm)_n_ film determined by ellipsometry (red) and AFM (black) before and after the jump. Error bars are shown in each graph, but when they are indistinguishable, it is because they are smaller than the point size.

When a data point was collected during the thickness jump (Figure 3a), the corresponding average thickness often displayed a large standard deviation (up to several tens of nm). This resulted from the coexistence of two distinct sets of thickness values. As described in Section 2.4, each reported thickness represents the average of ten measurements taken at random locations on the sample surface, at a few millimeters distance from each other. During the jump, some measurements correspond to regions exhibiting the prejump thickness, whereas others reflect the postjump thickness, with no intermediate values detected. These two groups of values are spatially distributed across the sample in an apparently random manner, which explains the elevated standard deviation. After the jump, films recovered a regular growth, and error bars returned to values comparable to those observed before the jump.

Ellipsometry measurements revealed a significant difference in film growth between tCNC_CV_- and tCNC_Lab_-based films prepared by dipping. Films composed of tCNC_CV_ exhibited a faster growth, with a thickness increment per LP of approximately 12 nm, compared to about 6 nm for tCNC_Lab_-based films. This difference was attributed to variations in particle dimensions and surface charge, which affected the amount of tCNC that adsorbed and stacked on the surface, leading to the formation of mikado-like films containing larger bundles and pores for tCNC_CV_ as confirmed by SEM analyses (Figure 4a,b).

**Figure 4:**
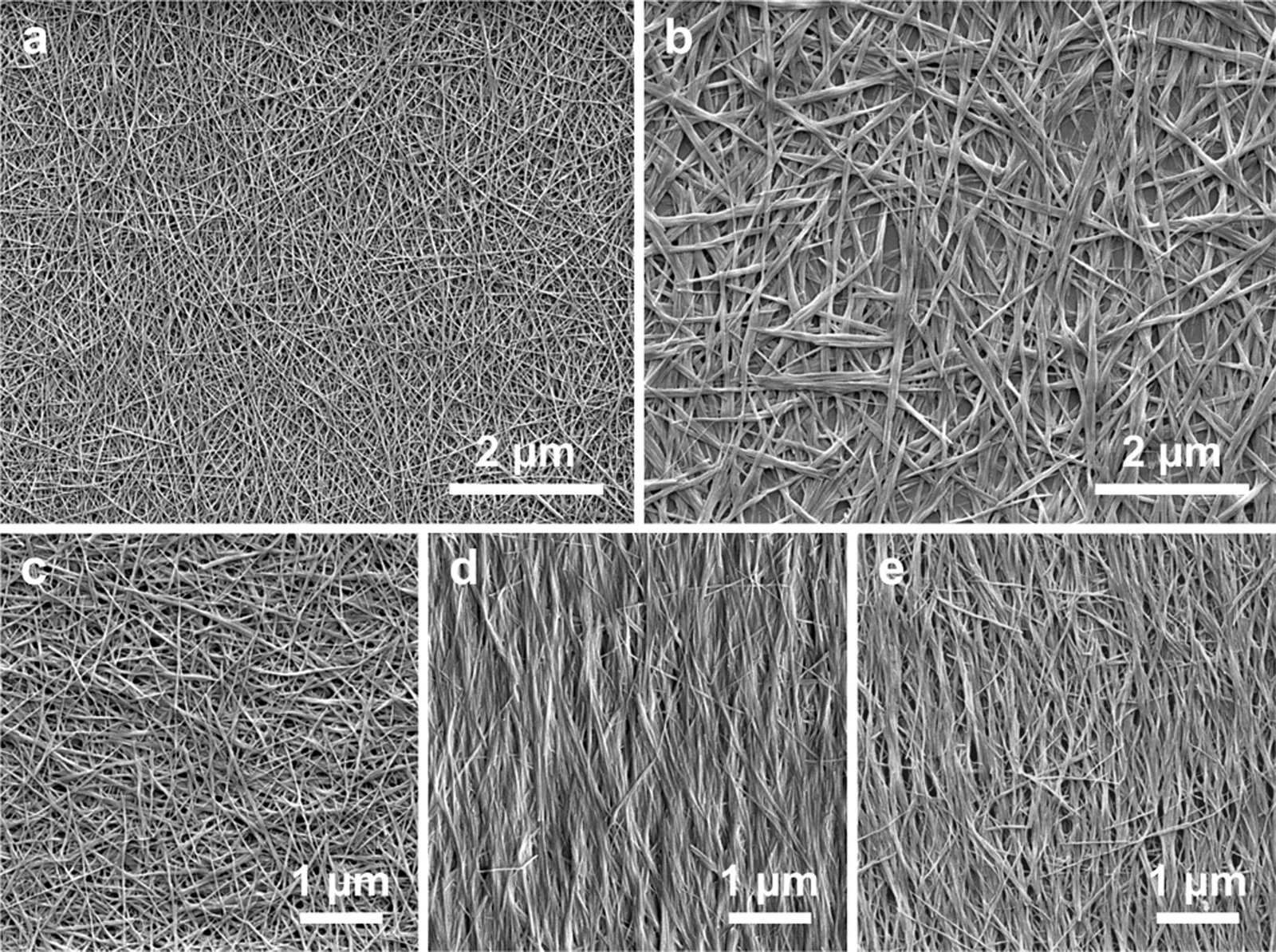
SEM images of (tCNC_Lab_/PVAm)_9_ (a) and (tCNC_CV_/PVAm)_7_ (b) films. SEM images of (tCNC_CV_/PVAm)_n_ films assembled by dipping ((c), *n* = 14), spin-assisted assembly ((d), *n* = 13), and grazing incidence spraying ((e), *n* = 16).

Specifically, tCNC_Lab_ exhibited an average particle height of 9 nm, whereas tCNC_CV_ reached approximately 25 nm. In an ideal situation, the thickness increment per LP is expected to roughly correspond to the combined thickness of the deposited tCNC particles and the adsorbed PVAm layer (a few nanometers). However, the increments measured by ellipsometry were consistently smaller than the anticipated values. Based on individual particle dimensions, the expected (tCNC/PVAm) LP thickness by dipping should be approximately 25 nm for tCNC_CV_ and 9 nm for tCNC_Lab_, whereas the experimental ellipsometric values were around 12 and 6 nm, respectively. This discrepancy most probably resulted from the laser spot size of the ellipsometer (∼1 mm^2^), averaging the optical contributions of both the tCNC material and the film porosity within the probed area, further contributing to an underestimation of the actual thickness. Moreover, the use of a fixed refractive index can introduce deviations between the expected and the measured thickness value.

Our results, consistent with earlier reports,^17,18^ indicate that the deposition of each (tCNC/PVAm) LP did not lead to a complete surface coverage. The tCNCs were randomly distributed, thus accumulated layers formed denser packing in the lower layers and sparser coverage in the upper layers (Figure 1). Consequently, the tCNC-based layers exhibited a porosity gradient along the out-of-plane direction. This interpretation was supported by the gradual increase in the refractive index from 1.1 to 1.4 during the film buildup, in agreement with previously reported data.^18^

To evaluate the contribution of each film component to the overall growth rate and the observed thickness jump, the ellipsometric thickness was measured after each individual layer deposition (Figure 3b). As expected, the total film growth rate was primarily governed by the tCNC layers, which exhibited an average thickness increment of approximately 10 nm per layer, whereas the polyelectrolyte (PVAm) layers contributed only modestly, with an increment of about 1–2 nm per layer. These results indicate that the atypical growth behavior originated from the deposition of the tCNC layer rather than from the deposition of the polyelectrolyte layer.

The deposition of additional layers resulted in more thickness jumps (Figure 3c), whose number and magnitude depended on the tCNC source. Specifically, two distinct jumps were observed for (tCNC/PVAm)_n_ films: at 7 and 12 LPs for the tCNC_CV_-based films, and at 11 and 21 LPs for the tCNC_Lab_-based films. The second jump was less pronounced in the tCNC_CV_-based film than in the tCNC_Lab_-based film, probably due to differences in aspect ratio, morphology, and surface charge, leading to the formation of a gradually less porous tCNC_CV_-based film.

However, such an unusual growth behavior has not been observed for multilayer films prepared with CNCs extracted from cotton and wood (cCNCs and wCNCs, respectively; see Figure S4), which possess a lower aspect ratio (12 and 14, respectively; Table S1) compared to tCNCs, or wCNFs, which, despite their high aspect ratio (40; Table S1), are more flexible than CNCs. These results strongly suggest that this structural transition is driven by the rigid rod-like character and the high aspect ratio of tCNCs organized within a porous mikado-like structure. Moreover, tCNC-based films are considerably more porous than those composed of cCNC-, wCNC-, or wCNF-based films due to their characteristic morphology, and this enhanced porosity may be the cause of the observed thickness jump.

All thickness values were measured by ellipsometry, an optical method that relies on a mathematical model, using a fixed refractive index of 1.465. It could therefore be argued that the observed sharp thickness transition might originate from an optical artifact inherent to the technique and to the mathematical fitting of the ellipsometric angles Δ and Ψ performed to retrieve the film thickness. To rule out this argument, we conducted complementary AFM measurements on samples collected before, during, and after the jump (Figure 3d, Figure S5). The AFM data verified the presence of the thickness jump, showing a growth rate consistent with that determined by ellipsometry before the transition. Interestingly, a clear discrepancy between AFM and ellipsometric thickness values was observed at 7 LPs and after the jump.

Although the origin of this difference remains unclear, a plausible explanation is that ellipsometry relies on an average refractive index that may deviate from the actual optical properties of the films, resulting in an underestimation of the true thickness. Ellipsometry probes a surface area of approximately 1 mm^2^, whereas AFM measures regions only a few tens of micrometers wide; such differences in sampling scale likely affect both the measured thickness and the associated standard deviation. Another plausible explanation for this thickness discrepancy is the partial detachment of the film during sample breaking to the appropriate dimensions (typically about 1 cm^2^) for AFM measurements, which may lead to an overestimation of the film thickness. Despite these limitations, the AFM results are sufficiently reliable to confirm the tendency revealed by ellipsometry results and that the observed thickness jump is not an optical artifact.

The spatial orientation of tCNCs within the (tCNC/PVAm)_n_ films assembled by dipping was investigated by Mueller matrix ellipsometry. Indeed, this technique was used in previous studies to confirm the preferential alignment of rod-shaped CNCs by dragging forces acting during layer deposition by dip-coating.^32^ Figure S6 shows the Mueller matrices measured for four different azimuthal orientations, obtained by rotating the sample about its normal. An azimuth of 0° corresponded to the configuration in which the sample withdrawal direction during dip-coating deposition lies within the plane of incidence. The Mueller matrix varied with the sample azimuth, revealing the presence of optical anisotropy. The matrix elements M_13_, M_23_, M_31_, M_32_, M_41_, and M_42_ were close to zero for azimuths of 0 and 90°. In contrast, these elements reached maximum values with opposite signs for azimuths of 45 and −45°. This behavior was consistent with a uniaxial or weakly biaxial anisotropic response, for which the optical axis was oriented either parallel or perpendicular to the plane of incidence at azimuths of 0 and 90°, respectively. The observed azimuthal dependence of the off-diagonal Mueller matrix elements further suggested that the anisotropy predominantly resulted from in-plane alignment effects. In particular, it indicated that the tCNCs were partially aligned within the plane of the sample, preferentially along the withdrawal axis of the dip-coating deposition, giving rise to a measurable optical anisotropy.

These results were used to study the origin of the thickness jump by conventional spectroscopic ellipsometry measurements at an azimuth of 0°. At each wavelength, two parameters, Is and Ic were measured. These parameters are related to the ellipsometric angles ψ and Δ through the following relations:

1 Is=sin(2ψ)sin(Δ)

2 Ic=sin(2ψ)cos(Δ)

Ellipsometric spectra measured for different numbers of deposited layers are shown in Figure S7. These spectra depend on the number of deposited layers. In order to analyze these spectra, it is necessary to build an optical model.

The optical model consists of a silicon substrate covered by an anisotropic layer composed of nanofibers perfectly aligned within the plane of incidence and voids. The ordinary (*ε_eff,o_*) and extraordinary (*ε*_*eff*,*e*_) dielectric functions are described using an anisotropic Bruggeman effective medium approximation:

3 $$f\frac{\varepsilon_{eff,o}-\varepsilon_{CNC}}{\varepsilon_{eff,o}+\varepsilon_{CNC}}+\left(1-f\right)\frac{\varepsilon_{eff,o}-1}{\varepsilon_{eff,o}+1}=0$$

4 $$\varepsilon_{eff,e}=f\varepsilon_{CNC}+1-f$$

*f* is the volume fraction of CNC and *ε_CNC_* its dielectric function. The free parameters are the volume fraction and film thickness. As shown in Figure S7, the model reproduces all measured spectra. The evolution of the CNC volume fraction as a function of the number of layer pairs is reported in Figure 5a,b. Initially, the cellulose volume fraction *f* increases with the number of layers, reaching a maximum at 11 layer pairs for the tCNC_Lab_ sample and 6 layer pairs for the tCNC_CV_ sample. The addition of a further layer leads to a drastic decrease in the volume fraction to reach a value of 35–40%. This decrease occurs concurrently with a steep increase in the layer thickness. This behavior suggests that the step increase in thickness is associated with an increase in the film porosity. This variation is accompanied by a decrease in the effective ordinary and extraordinary refractive indices of the film (Figure 5c,d), in accordance with eqs 3 and 4.

**Figure 5:**
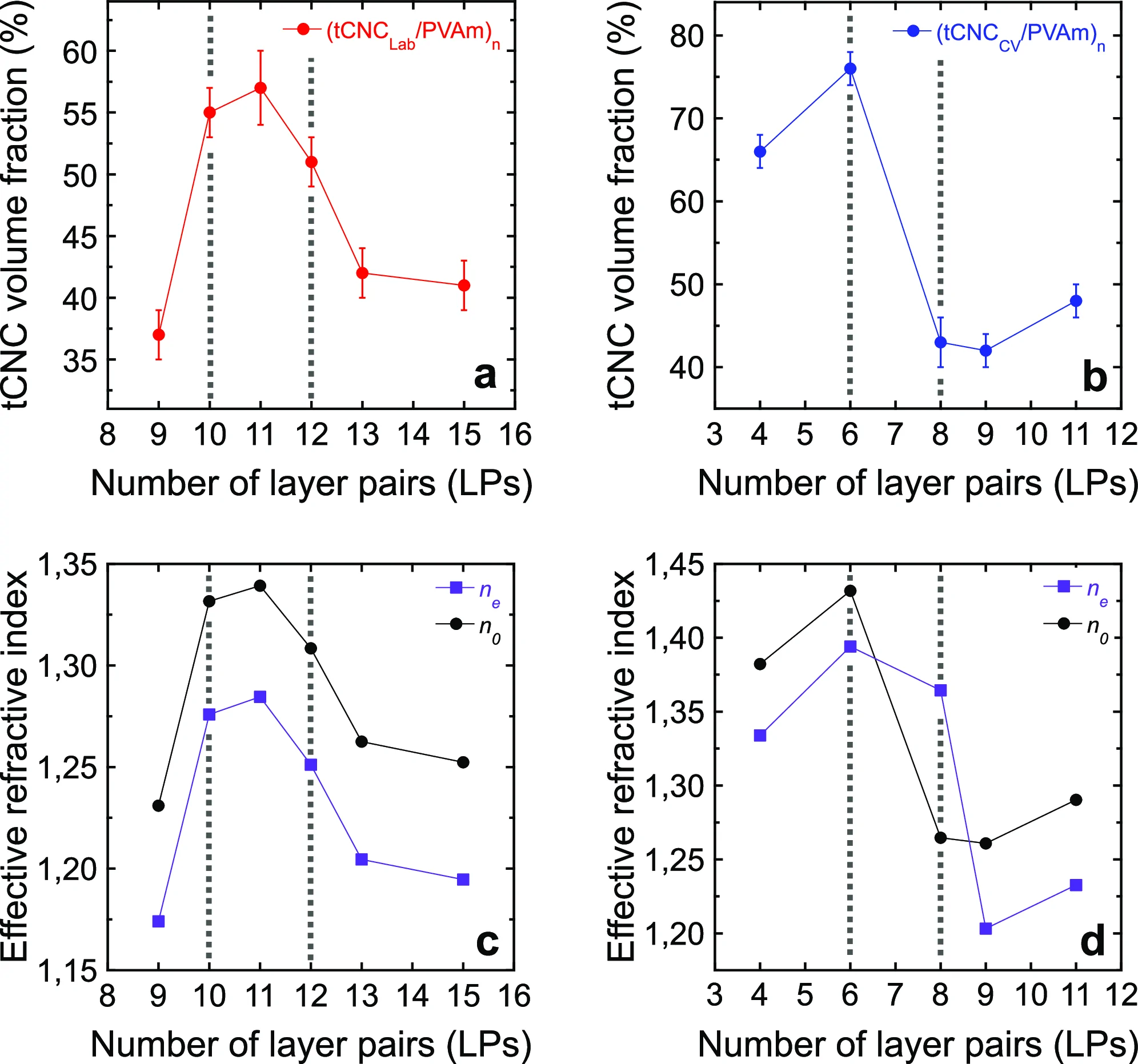
Variation of the volume fraction of tCNCs (a), (b) and ordinary (n_o_, black) and extraordinary (n_e_, green) effective refractive indices (c), (d) of (tCNC_Lab_/PVAm)_n_ (a), (c) and (tCNC_CV_/PVAm)_n_ (b), (d) films prepared by dipping. The dotted lines outline the area where the thickness jump occurs.

### 3.2 Influence of Deposition Conditions on the Thickness Jump

Multilayered films composed of alternating layers of CNCs and PVAm were fabricated using LbL assembly. We investigated seven parameters that can influence the film buildup and thus the thickness jump, namely the deposition method, substrate nature, CNC concentration, ionic strength, and pH of the PVAm solution, type of polycation, and nature of the interactions between the polymer and CNCs.

### 3.3 Influence of the Deposition Method

We first examined the effect of the assembly method on nanocomposites composed of tCNC_CV_ and tCNC_Lab_ (Figure 6a,b). Films were fabricated by spray- and spin-assisted assembly using the same tCNC and PVAm solutions as in the dipping process. A thickness jump at approximately 50–60 nm during the film buildup was observed regardless of the deposition method, although it was significantly less pronounced for spin-assisted LbL films. This behavior was attributed to the centrifugal forces inherent to spin-coating, which promoted the radial in-plane alignment of tCNCs, leading to the formation of denser layers with lower porosity compared to those obtained by dipping and orthogonal spraying (Figure 4c,d).^33^

**Figure 6:**
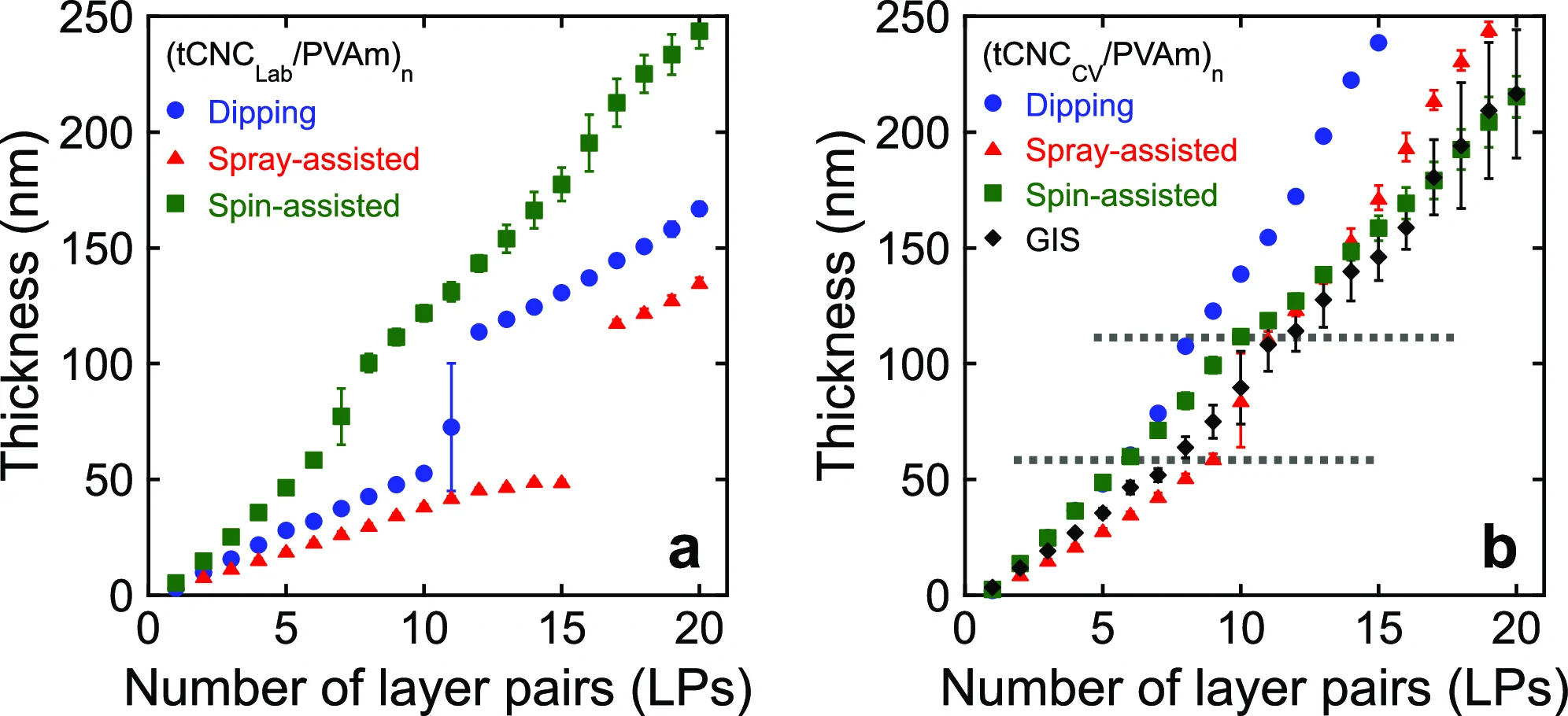
Variation of the thickness of (tCNC_Lab_/PVAm)_n_ (a) and (tCNC_CV_/PVAm)_n_ (b) films prepared by dipping (blue), spray-assisted assembly (red), spin-assisted assembly (green) and grazing incidence spraying (black) as a function of the number of layer pairs monitored by ellipsometry.

For the tCNC_CV_-based nanocomposites (Figure 6b), the dipping and spin-assisted methods initially showed a higher growth rate, with a thickness increment per LP of about 10 nm, compared to about 6.3 nm for the spray-assisted method. However, after approximately 10 LPs, the unusual increase in growth caused the orthogonal spray method to surpass spin-assisted assembly.

Surprisingly, the behavior of the tCNC_Lab_-based nanocomposite (Figure 6a) assembled with different LbL methods was different. The spin-assisted method produced the highest growth rate, with a thickness increment per LP of about 10 nm before the jump, followed by dipping (about 5 nm per LP before the jump), while the orthogonal spray-assisted method yielded the lowest growth rate (about 3.8 nm per LP before the jump). This behavior was attributed to the reduced film porosity induced by the in-plane alignment of tCNCs within each layer, resulting in a denser film structure.

The observed growth differences are consistent with literature reports for conventional polyelectrolyte multilayers and are inherent to the specific conditions of each deposition method.^23,34,35^ In general, spray-assisted assembly produces thinner films and spin-assisted assembly produces thicker ones than dipping. In dipping, a “mikado-like” arrangement is typically observed,^17,18^ where tCNCs are stacked in a more random fashion, creating larger voids. The densification effect induced by spin-coating is expected to be more pronounced for tCNC_Lab_ than for tCNC_CV_ due to the higher aspect ratio of tCNC_Lab_, which can explain the higher difference in growth rates between spin-coated and dip-coated films in the case of tCNC_Lab_. Despite these discrepancies in growth trends, the growth rates of spin-coated films prepared from tCNC_CV_ and tCNC_Lab_ were nearly identical, with a thickness increment of approximately 10 nm per LP before the jump. We assumed that for films containing highly anisometric nanoparticles, the rinsing under high shear conditions during spin-coating promoted the desorption of weakly bound tCNCs, which would not be rinsed off in the case of dipping.^25,33^ It should also be noted that the dipped films were prepared by immersion of the substrate in the tCNC_CV_ suspension or PVAm solution for 5 min, rather than for only a few seconds. This fact alone could explain the growth difference for tCNC_CV_-based films assembled by dipping and spin-assisted assembly.

As for tCNC-based films prepared by spin-assisted assembly, the thickness jump was significantly less pronounced for films assembled by GIS due to the alignment of CNCs and the formation of a denser structure (Figure 4e). The fact that GIS-assisted films were thinner than those fabricated by spin-assisted assembly came from variations in the surface density and spatial organization of nanocellulose in each layer, which is known to be specific to each deposition method.

### 3.4 Influence of the Substrate Nature

To investigate the impact of the substrate on the occurrence of the thickness jump, we prepared samples in which several PSS/PAH layer pairs were deposited prior to the assembly of a (tCNC_CV_/PVAm)_n_ film (Figure S8). (PSS/PAH)_m_ multilayers are known to form uniform and dense films.^4^ By varying the number of (PSS/PAH) layer pairs, we introduced a spacer between the underlying substrate and the subsequently deposited (tCNC_CV_/PVAm)_n_ film. The inclusion of a (PSS/PAH)_50_ underlayer beneath a (tCNC_CV_/PVAm)_10_ film did not significantly alter the growth behavior. Since the thickness of (PSS/PAH)_50_ was relatively small (10.4 ± 0.2 nm), the growth profile of (PSS/PAH)_50_(tCNC_CV_/PVAm)_10_ film closely followed that of the (tCNC/PVAm)_10_ film, exhibiting a jump of nearly the same amplitude, shifted by one LP. In contrast, when a thicker (PSS/PAH)_150_ film was used as an underlying layer, the subsequent (tCNC_CV_/PVAm)_10_ film displayed a linear growth with a less pronounced jump. For all three multilayered nanocomposites, the characteristic thickness jump was present. Although it appeared at different LP numbers, it consistently corresponded to approximately 50–60 nm of (tCNC_CV_/PVAm)_n_ film thickness. For instance, in the (PSS/PAH)_50_(tCNC_CV_/PVAm)_10_ sample, the jump occurred at a thickness of about 70 nm, comprising around 10 nm from the 50 (PSS/PAH) LPs and around 60 nm from the (tCNC_CV_/PVAm)_10_ film. However, the magnitude of the jump clearly became less pronounced with increasing thickness of the underlying (PSS/PAH)_m_ multilayer. These results indicate that the substrate surface had no significant effect on the presence of the jump, as it remained visible when the tCNC-based films were assembled on (PSS/PAH)_m_ films.

### 3.5 Influence of tCNC Concentration

It is also well established that the concentration of CNCs influences the growth of CNC-based multilayer films.^36^ Specifically, increasing the CNC concentration leads to a higher film growth rate. We explored whether the tCNC concentration also affects the amplitude and the occurrence of the thickness jump, in addition to its impact on the growth rate. tCNC-based LbL films were prepared by dipping using tCNC_CV_ and tCNC_Lab_ suspensions at concentrations of 0.1, 0.2, 0.4, and 0.8 g·L^–1^ (Figure 7a,b).

**Figure 7:**
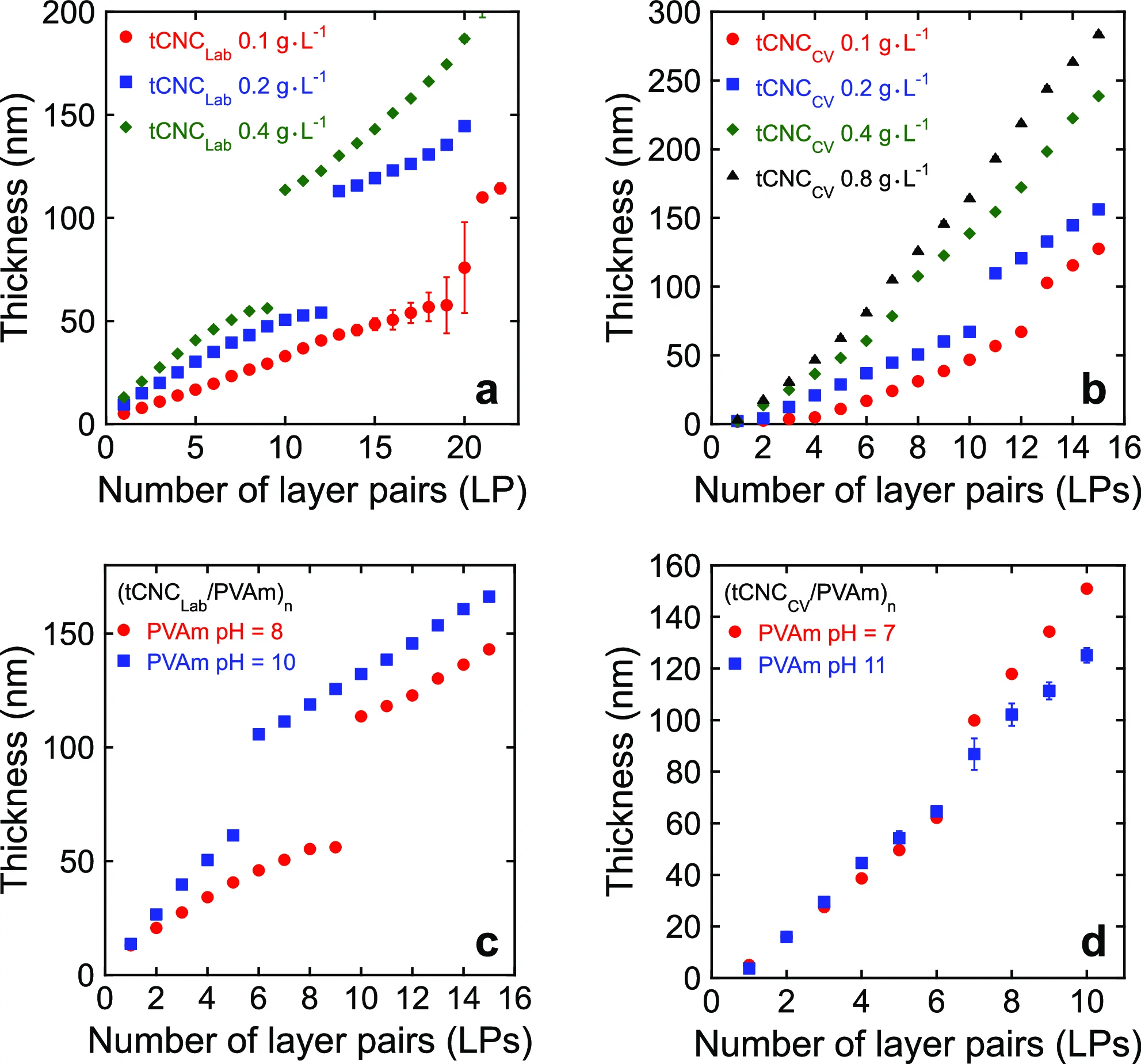
Variation of the thickness of (tCNC_Lab_/PVAm)_n_ (a) and (tCNC_CV_/PVAm)_n_ (b) films assembled by dipping with various concentrations of tCNCs (0.1 (red), 0.2 (blue), 0.4 (green) and 0.8 (black) g·L^–1^) as a function of the number of layer pairs monitored by ellipsometry. (c) Variation of the thickness of tCNC_Lab_-based films prepared by dipping as a function of the pH of the PVAm solution (pH = 8 (red) or 10 (blue) and the number of layer pairs monitored by ellipsometry. (d) Variation of the thickness of tCNC_CV_-based films prepared by dipping as a function of the pH of the PVAm solution (pH = 7 (red) or 11 (blue) for tCNC) and the number of layer pairs monitored by ellipsometry. Error bars are shown in each graph, but when they are indistinguishable, it is because they are smaller than the point size.

As expected, the growth rate depended on the tCNC concentration, which altered the number of layer pairs at which the unexpected jump occurs. Indeed, an increase in tCNC concentration from 0.1 to 0.4 g·L^–1^ induced an increase in the film growth and shifted the thickness jump to lower LPs. A thickness jump at around 50–60 nm was consistently observed for all tCNC_CV_ and tCNC_Lab_ concentrations. However, in contrast to (tCNC_Lab_/PVAm)_n_ films, the amplitude of the jump for (tCNC_CV_/PVAm)_n_ films was more pronounced at lower concentrations and nearly disappeared at higher suspension concentrations. This behavior can be attributed to the greater amount of tCNCs deposited on the surface at higher concentrations, allowing the critical thickness transition to be reached with a lower LP number and resulting in the formation of less porous films. Although the critical number of LPs varied, the thickness jump consistently occurred within the 50–110 nm range. At low tCNC_Lab_ concentrations, it was even possible to get thickness measurements during the jump, showing a progressive increase of the standard deviation that strongly reduced after the jump. Surprisingly, at a tCNC_CV_ concentration of 0.8 g·L^–1^, the film growth was somewhat similar to the one observed at 0.4 g·L^–1^, but the thickness jump was not visible anymore. Instead, the growth curve was decomposed into two linear regimes intersecting in the region where the thickness transition occurred, strongly suggesting the formation of a less porous film.

### 3.6 Influence of the pH of the PVAm Solution

The pH of the polycation solution is another parameter known to influence the growth rate of nanocellulose-based multilayer films.^37^ For weak polycations, such as the PVAm used in this work, increasing the pH reduces the degree of protonation and thereby decreases the surface charge density of the polyelectrolyte. Weak polyelectrolytes typically adopt an extended chain conformation when highly charged, but as the pH increases and charge density decreases, the polymer chains transition to a coil-like conformation. This change in conformation and surface charge density influences the film growth rate. Merindol et al.^37^ reported an increase in the growth rate of (CNF/PVAm)_n_ multilayers when the pH varied from 8 to 11. Typically, as the pH of the PVAm solution increases, the film buildup speed increases due to an increase in the amount of PVAm adsorbing on the surface to compensate for the nanocellulose charges. We investigated how the pH of the PVAm solution affected the growth of nanocellulose-based films, as well as the amplitude and appearance of the thickness jump during film formation. Various (nanocellulose/PVAm) films were built by dipping with a PVAm solution at various pH and the growth of the multilayer films was monitored by ellipsometry (Figure 7c,d, Figure S9, and Figure S10). The pH and nanocellulose source (tCNC_Lab_, tCNC_CV_, cCNCs, wCNCs and wCNFs) were varied while the other conditions were kept fixed. As expected, the growth rates of (cCNC/PVAm)_n_, (wCNC/PVAm)_n_ and (wCNF/PVAm)_n_ films increase with increasing pH of the PVAm solution and no thickness jump could be observed. For pH values around 10, which corresponds to the p*K*_a_ of the PVAm,^38,39^ the surface charge density decreases, resulting in a change of conformation of PVAm, which induced an increase in the thickness for each LP. Please note that the p*K*_a_ value of a polyelectrolyte is not a single fixed value and may vary by 1–2 orders of magnitude depending on the chemical environment, ionic strength, solvent conditions, and polymer conformation. Electrostatic interactions along the polymer chain can indeed modify the protonation behavior of the ionizable groups, leading to an apparent p*K*_a_ that differs from that of the corresponding monomers. Surprisingly, the growth rate of the (tCNC_CV_/PVAm)_n_ film appeared to be independent of the pH of the PVAm solution, whereas a pH-dependent behavior was observed for films constructed with tCNC_Lab_. In the latter case, the thickness jump appeared earlier when the pH of the PVAm solution increased. This behavior was similar to the one observed with an increasing concentration of tCNCs for a given pH, as depicted in Figure 7a,b. This difference was therefore attributed to the distinct type of nanocellulose and the specific tCNC morphology (size and surface charge), which were defined by the preparation process. In addition, it should be noted that tCNCs are significantly larger than the PVAm chains. Although PVAm underwent a conformational transition from an extended state to a coil-like state, its characteristic dimensions remained far smaller than those of the tCNCs. However, the impact of this conformational change on tCNC film growth is not well understood, and the growth rate seemed to be primarily governed by the size of the tCNCs. Notably, the unusual thickness jump was still observed for the (tCNC/PVAm)_n_ films, regardless of the pH of the PVAm solution. Consequently, we concluded that the jump was independent of the conformation and surface charge density of PVAm.

### 3.7 Influence of the Ionic Strength of the PVAm Solution

Another parameter known to influence the growth rate of LbL films is the ionic strength.^4,40,41^ The addition of salt to the polyelectrolyte solution screens the charges, enabling a conformational transition of the polymer chains from extended to coil-like, similar to the effect observed upon increasing pH.

Screening electrostatic interactions along the polymer chain usually makes the apparent p*K*_a_ shift toward the intrinsic p*K*_a_ of the individual ionizable groups, because charge/charge repulsion within the polymer is reduced. Indeed, we measured a pH around 8 for PVAm in Milli-Q water and a pH around 9 for PVAm in 1 M NaCl. This reduction of the effective charge of the PVAm by increasing the ionic strength leads to (1) a conformational change of the polymer chain from extended to coiled, leading to the deposition of higher amounts of PVAM to compensate the nanocellulose charges and (2) a weakening of the interactions between PVAm and nanocelluloses, although at high concentrations, it may lead to film delamination. In the present study, the addition to the PVAm solution of NaCl at different concentrations resulted in variations in the growth rate of the (tCNC_CV_/PVAm)_n_ films, although the thickness jump was observed regardless of the salt concentration (Figure 8a).

**Figure 8:**
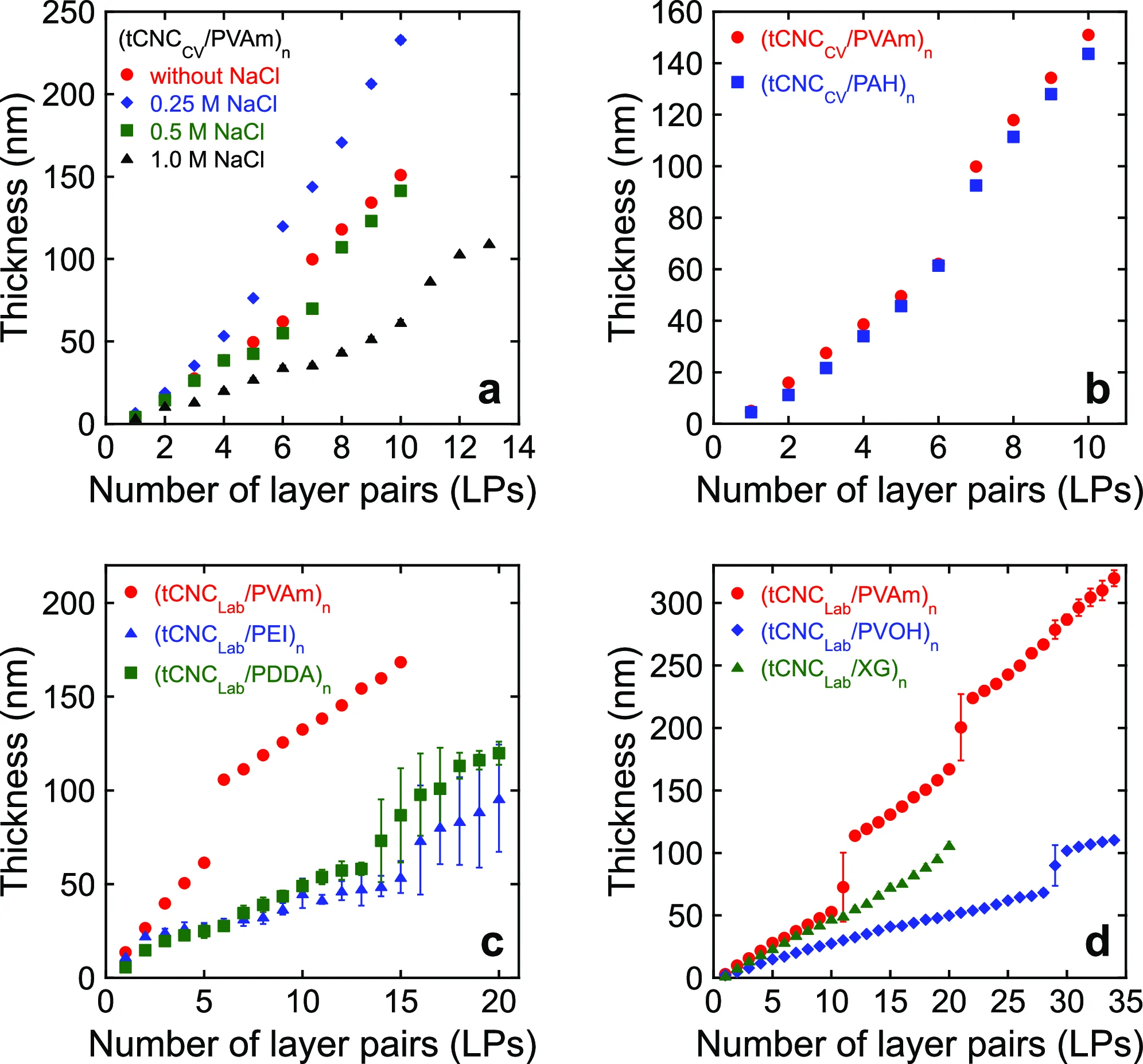
a) Variation of the thickness of tCNC_CV_-based films assembled with PVAm solution in the absence of salt (red), and in the presence of 0.25 M (blue), 0.5 M (green), and 1 M (black) NaCl as a function of the number of layer pairs. b) Variation of the thickness of tCNC_CV_-based films prepared with different polyelectrolytes such as PVAm (red) and PAH (blue) as a function of the number of layer pairs. c) Variation of the thickness of tCNC_Lab_-based films prepared by dipping with different polycations (PEI (blue), PVAm (red), and PDDA (green)) as a function of the number of layer pairs. Please note that large error bars were measured for films made of PEI after the jump. d) Variation of the thickness of tCNC_Lab_-based films prepared by dipping with a charged (PVAm (red)) and uncharged polymers (PVA (blue), xyloglucan (green)) as a function of the number of layer pairs. Error bars are shown in each graph, but when they are indistinguishable, it is because they are smaller than the point size.

Ellipsometry measurements revealed an increased growth rate of films assembled in the presence of 0.25 M NaCl in the PVAm solution. Similar to the effect of a pH increase, the conformational change induced by adding 0.25 M NaCl in the PVAm solution accelerated the growth rate. However, when the NaCl concentration in the PVAm solution was further increased (0.5 and 1 M), the growth rate decreased. Conformational change of the PVAm chains and interaction weakening are responsible for the changes in the growth rate observed in Figure 8a where the ionic strength in the PVAm solution was increased. When exposing LbL films to higher salt concentrations during the buildup process, swelling of the films occurs.^40,42^ Indeed, salt diffuses from the solution into the film in order to reach an equilibrium state between the salt ions in the solution and the ones in the film. The salt diffusing in the film interacts with the charged species by screening the charges between the oppositely charged molecules resulting in the formation of extrinsic compensation instead of intrinsic compensation (corresponding to a decrease in the entropy gain). Therefore, the ionic bonds between the oppositely charged moieties are weakened and the mobility of the charges species is enhanced. If enough ionic bonds are broken, the LbL film dissolves into individual components in solution or into complexes. However, if the film is in a swollen state without undergoing a dissolution process, the replacement of the salt medium with water (rinsing step) leads to the expulsion of the salt from the film. As a result, the ionic bonds between the oppositely charged species will be reformed. In this study, LbL growth was observed regardless of salt concentration, suggesting that, at high salt concentrations, the lower growth rate may be due to a smaller amount of material deposited on the surface. In addition, the data clearly show that the jump is not affected by the salt concentration. As for pH and concentration, its occurrence is just shifted to the corresponding LP where the critical thickness (50–60 nm) is reached.

### 3.8 Influence of the Polycation Nature

We studied the influence of the polycation nature on the film growth and thickness jump. tCNC-based multilayer films were prepared using other polycations, such as PEI (a weak branched polyelectrolyte), PAH (a weak linear polyelectrolyte), and PDDA (a polyelectrolyte insensitive to pH) instead of PVAm (Figure 8b,c). LbL films composed of tCNC_Lab_ and PEI or PDDA were prepared by dipping under the same conditions used for PVAm. A lower growth rate was observed for films composed of CNC_Lab_ and PDDA or PEI compared to those assembled with PVAm, and a thickness jump with a reduced amplitude was visible after 14 and 15 deposition cycles, respectively, compared to only 5 with PVAm (Figure 8c). In contrast, films made of tCNC_CV_ and PVAm, or PAH, exhibited a similar growth rate (Figure 8b). We also tried to assemble tCNC_CV_ with PEI, but the resulting growth was very low. Therefore, we did not further investigate the building of (tCNC_CV_/PEI)_n_ nanocomposites, since studying the jump in this case would require a large number of deposition cycles. The growth discrepancy observed between films built with tCNC_Lab_ and tCNC_CV_ was attributed solely to differences in intrinsic characteristics (e.g., aspect ratio, surface charge density) of tCNCs used in these films with the respective polyelectrolytes. Thus, the thickness jump was not related to the type of polyelectrolyte.

### 3.9 Influence of the tCNC/Polymer Interaction Type

To explore the influence of the interaction type on the film growth and thickness jump, we fabricated tCNC_Lab_-based films with neutral synthetic and natural polymers by dipping under the same conditions used for the (tCNC_Lab_/PVAm)_n_ films. PVA and XG were chosen as they were previously used to assemble LbL films via hydrogen bonding (Figure 8d).^36,43–45^ In hydrogen-bonded LbL films, hydrogen bond formation typically displaces water molecules that were hydrogen-bonded to the polymer chains, so water release is driving the assembly process. That water expulsion contributes favorably to the free energy of film formation, because the new polymer–polymer hydrogen bonds are preferred over polymer–water interactions under the assembly conditions. In that sense, water release is not just a side effect; it is one of the thermodynamic reasons why hydrogen-bonded multilayers can form.

Ellipsometry measurements for (tCNC_Lab_/PVA)_n_ films showed a linear growth accompanied by the unexpected jump. However, the growth rate for (tCNC_Lab_/PVA)_n_ films was significantly lower than that of the (tCNC_Lab_/PVAm)_n_ films, indicating that more layer pairs were required to reach the critical thickness at which the thickness jump occurs. In addition, the amplitude of the jump was smaller for films assembled by hydrogen bonding than for those built using electrostatic interactions. The presence of the unusual growth in both (tCNC_Lab_/PVAm)_n_ and (tCNC_Lab_/PVA)_n_ suggests that the thickness jump was independent of the type of interaction between the tCNCs and the polymer matrix. However, the assembly of tCNC_CV_ with XG, showed a regular growth rate with a small fluctuation between approximately 40 and 60 nm. Despite this local variation, the general growth rate remained linear, so attributing this variation to the thickness jump observed with PVAm and PVA is difficult. These results allowed us to conclude that the occurrence of the thickness jump was not linked to the electrostatic interactions between the tCNCs and polyelectrolyte.

### 3.10 Possible Origin of the Thickness Jump during the Buildup of tCNC-Based Films

Data acquired in this work suggest that the occurrence of the thickness jump was not governed by the deposition conditions (e.g., tCNC concentration, nature of polycation, nature of interactions, ionic strength, pH of PVAm solution) but rather resulted from the intrinsic properties of tCNCs (e.g., aspect ratio, surface charge density) and their organization in the film, as it was never observed for films built with both shorter CNCs extracted from cotton or wood and wCNFs in conjunction with PVAm. It should also be noted that the jump always occurred approximately at the same film thickness (50–60 nm) but at a different number of deposited LPs depending on the suspensions and deposition technique and conditions. To the best of our knowledge, this phenomenon has not previously been reported in the literature. In light of the various studies on the jump, several possible explanations can be considered, each requiring further experimental verifications to be confirmed or ruled out.

The spontaneous deposition of a larger amount of tCNCs within a specific LP appears counterintuitive, since all deposition steps during the multilayer buildup were carried out under identical conditions.

The most likely scenario is that the progressive stacking of randomly organized tCNC layers generated mechanical stress within a 3D porous film.^46^ Results suggest that the observed transition cannot occur from the tension that was built up during the first deposition/drying cycles. The initial structure was driven by the kinetics of the deposition process and locked into place until the transition occurred. When more layers were deposited onto a postjump structure, they adopted again the kinetically more stable prejump structure until the next jump-transition occurred. This suggests that each prejump structure was temporarily stabilized during growth and can only rearrange and expand its volume through the rupture of crosslinking points within the 3D-network of rigid cellulosic rods. The accumulated stress during the film buildup arose from the fact that the formed structure resulted from a kinetic mechanism that does not allow the system to reach thermodynamic equilibrium, which it then attempted to attain through the jump. This tension release led to the formation of a more equilibrated, less dense structure. This interpretation is supported by the ellipsometric measurements showing a drastic decrease in the tCNC volume fraction associated with an increase in the film porosity. Unfortunately, SEM images of the sample surface before, during, and after the jump did not reveal noticeable differences in porosity. However, at the same magnification, clear morphological differences were observed between tCNC_Lab_ and tCNC_CV_-based nanocomposites: the former exhibited more aggregates and bundles, whereas the latter displayed stacks of thinner, more individualized nano-objects (Figure 4a,b). In addition, it is clear that the jump-transition requires long objects to couple collective actions over long distances more efficiently. This argument is in agreement with the fact that films composed of very short cellulosic building blocks did not show such jump transitions.

Alternatively, the accumulation and subsequent release of electrical charges within the film could also contribute to the phenomenon, with the local increase in surface charge density facilitating the deposition of a larger amount of tCNCs at a specific thickness.

Finally, we considered the possibility that tCNCs adopt a more vertical position when deposited at a specific porosity, corresponding to the thickness range at which the jump takes place (50–60 nm). However, it is difficult to imagine how micrometer-long objects could penetrate a rigid porous network of only 50–60 nm in thickness and induce only a doubling of the film volume in the vertical direction (corresponding to 100–120 nm in thickness). AFM analyses of tCNC-based films performed before and after the jump in the dry state showed no morphological difference. It remains conceivable that a more vertical positioning of the tCNCs could occur at a given porosity in the wet state, followed by their reorientation (horizontal flattening) during drying. However, this assumption could not be verified, as the drying step is essential for the formation of tCNC-based films.

## 4 Conclusion

We reported on the discovery of a sharp thickness transition during the fabrication of multilayer films composed of tCNCs and PVAm monitored by ellipsometry. The AFM analysis supported the results obtained by ellipsometry and ruled out any optical artifact due to ellipsometry. Systematic ellipsometric measurements revealed that this sharp transition in thickness was unaffected by the deposition method, the nature of the polycation, the CNC concentration, the PVAm solution pH, or the nature of CNC/polymer interaction. These parameters only influenced the LP number at which the transition occurs, typically at a thickness of around 50–60 nm. Instead, this phenomenon appeared to be intrinsically linked to the high aspect ratio, the rigidity, and dimensions of tCNCs, as it was not observed in LbL films composed of low-aspect-ratio CNCs or CNFs. Ellipsometry measurements further showed the fabrication of anisotropic (tCNC_Lab_/PVAm)_n_ films by dipping, which arises from the preferential in-plane alignment of tCNCs induced by dragging forces during film deposition. The observed discontinuity in film thickness was clearly associated with a pronounced reduction in the tCNC volume fraction accompanied by a rapid expansion of the porous volume. Ellipsometry and AFM data suggested the formation of a highly unusual nonequilibrium film structure before the jump transition. The initial structure would have been driven by the kinetics of the deposition process and locked into place until the transition occurred. When additional layers were deposited on the surface of a postjump film, they would have adopted again the kinetically more stable prejump structure until the next transition occurred. It means that the postjump structure was thermodynamically more stable, and that the kinetically favored prejump structure only collapsed when the volume of the film was at a critical value at which the deposition conditions generated sufficient instabilities in the bulk film so that the transition could be triggered. One may speculate that the initial prejump structure is temporarily stabilized because rigid-rod 3D-networks (mikado-like structures) cannot rearrange and expand their volume without stretching of the rigid cellulosic rods (which is impossible) or without breaking crosslinking points in the network. In the present case we believe that the structural transition at the jump comes about by breaking crosslinks which releases the accumulated tension while adopting a more equilibrated less dense structure. This argument is in agreement with the fact that films composed of very short cellulosic building blocks do not show such jump transitions. We therefore suggest that the jump transition is related to an affine volume expansion which is used to describe deformations in (gel-like) 3D fiber networks where the only allowed motion is through rigid-body translation/rotation and motion at crosslinks. The system that we have constructed and discussed in the present manuscript belongs to a group of nanocomposite materials with a mikado-like microstructure.^47^ While such composites are typically volume materials resulting from assembly dynamics in bulk, our system is constructed step-by-step and allows to fine-tune the material composition and microstructure of mikado-networks. Despite extensive experimental investigations, the underlying mechanism responsible for this phenomenon remains unclear. Ongoing studies focus on the film growth process and internal structure using complementary techniques and aim to provide deeper insight into the origin of this unexpected phenomenon.

## Supplementary Material



## Data Availability

Data will be made available upon request to the corresponding authors.

## Notes

Nanocellulose morphology, dimensions, and aspect ratio; influence of the dipping time on film growth; influence of the nanocellulose nature on film growth; film thickness measurement using AFM; ellipsometric spectra measured for different numbers of deposited layers; influence of the substrate nature; influence of the pH of the PVAm solution on film growth (PDF)

## #

ENS de Lyon, CNRS, LCH, UMR5182, F-69342 Lyon, France
