# Supplementary material for: Unexpected Thickness-Driven Volume Expansion in Tunicate Cellulose Nanocrystal-Based Multilayer Films
Source: ACS Mater Au. 2026 Aug 21;6(6):1324–36. doi: 10.1021/acsmaterialsau.6c00047 (PMC13760640; doi:10.1021/acsmaterialsau.6c00047)
Supplement: Supplementary file 1 [file mg6c00047_si_001.pdf]

Supporting Information

Unexpected Thickness-Driven Volume Expansion in Tunicate Cellulose Nanocrystal-Based Multilayer Films

Leïla Garnier [a], Jean Muller [a], Pelinsu Demir [a], Labonno Azad [a], Pavithra Wijeratne [a], Christophe Contal [a], Christine Lancelon-Pin [b], Michel Tschopp [a], Yann Battie [c], Gero Decher [a,d], Matthias Pauly [a,#], Jean-Luc Putaux [b], Bruno Jean [b]*, and Olivier Felix [a,d]*

[a] *Université de Strasbourg, CNRS, Institut Charles Sadron UPR22, F-67000 Strasbourg, France*

[b] *Univ. Grenoble Alpes, CNRS, CERMAV, F-38000 Grenoble, France*

[c] *Université de Lorraine, LCP-A2MC, F-57000 Metz, France*

[d] *International Center for Frontier Research in Chemistry. F-67083 Strasbourg, France*

[#] *Present Address: ENS de Lyon, CNRS, LCH, UMR5182, F-69342 Lyon, France*

* E-mail: olivier.felix@ics-cnrs.unistra.fr

* E-mail: bruno.jean@cermav.cnrs.fr

1. Nanocellulose morphology, dimensions and aspect ratio

The TEM images in Figure S1 illustrate the differences in morphology and size of $tCNC_{CV}$, cCNC, and wCNF samples. $tCNC_{CV}$ are a few µm-long rodlike particles composed of a few laterally associated crystallites. cCNCs are also composed of a few crystallites, but they are significantly shorter (150-250 nm). The morphology of wCNFs is more difficult to describe as the individual elements were entangled and kinked during the extraction and purification treatments. They form loose networks that could be fully disrupted by mild sonication.

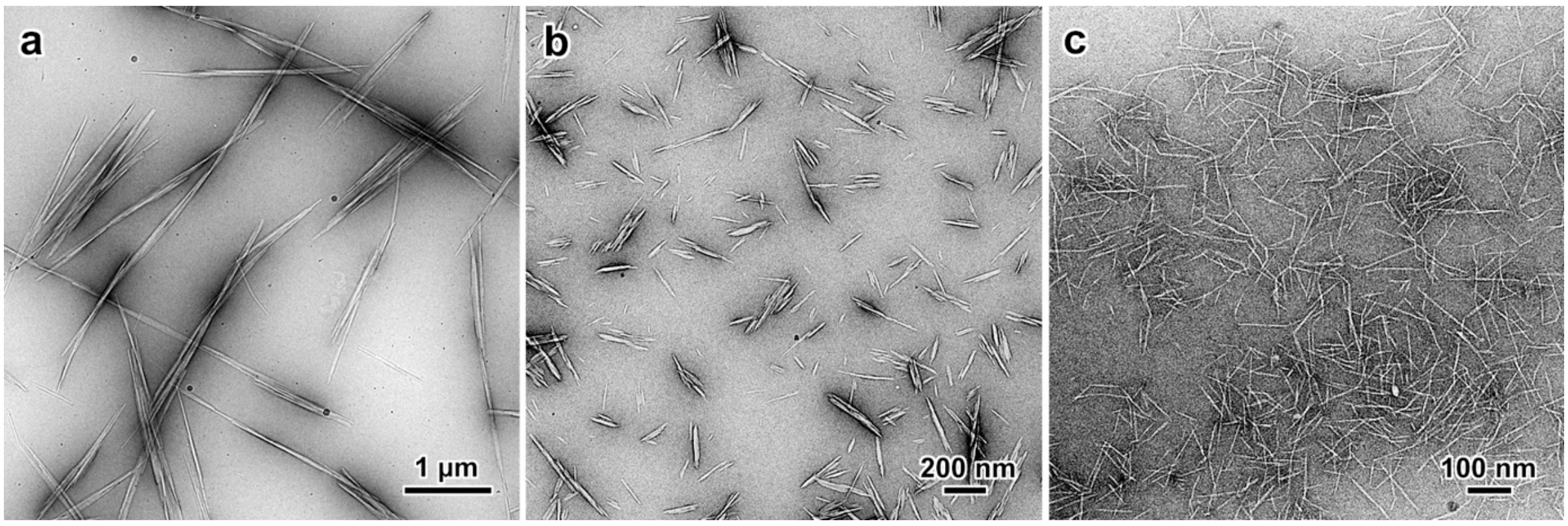

Figure S1. *TEM images of negatively stained preparations from $tCNC_{CV}$ (a), cCNC (b), and wCNF (c) suspensions.*

AFM images of submonolayers (dispersions prepared by dipping in a dilute suspension) of nanocelluloses deposited by dipping onto PEI-coated silicon wafers were analyzed to characterize the dimensions of nanocelluloses used in this study ($tCNC_{CV}$, $tCNC_{Lab}$, cCNCs, wCNCs and wCNFs) (**Figure S2**).

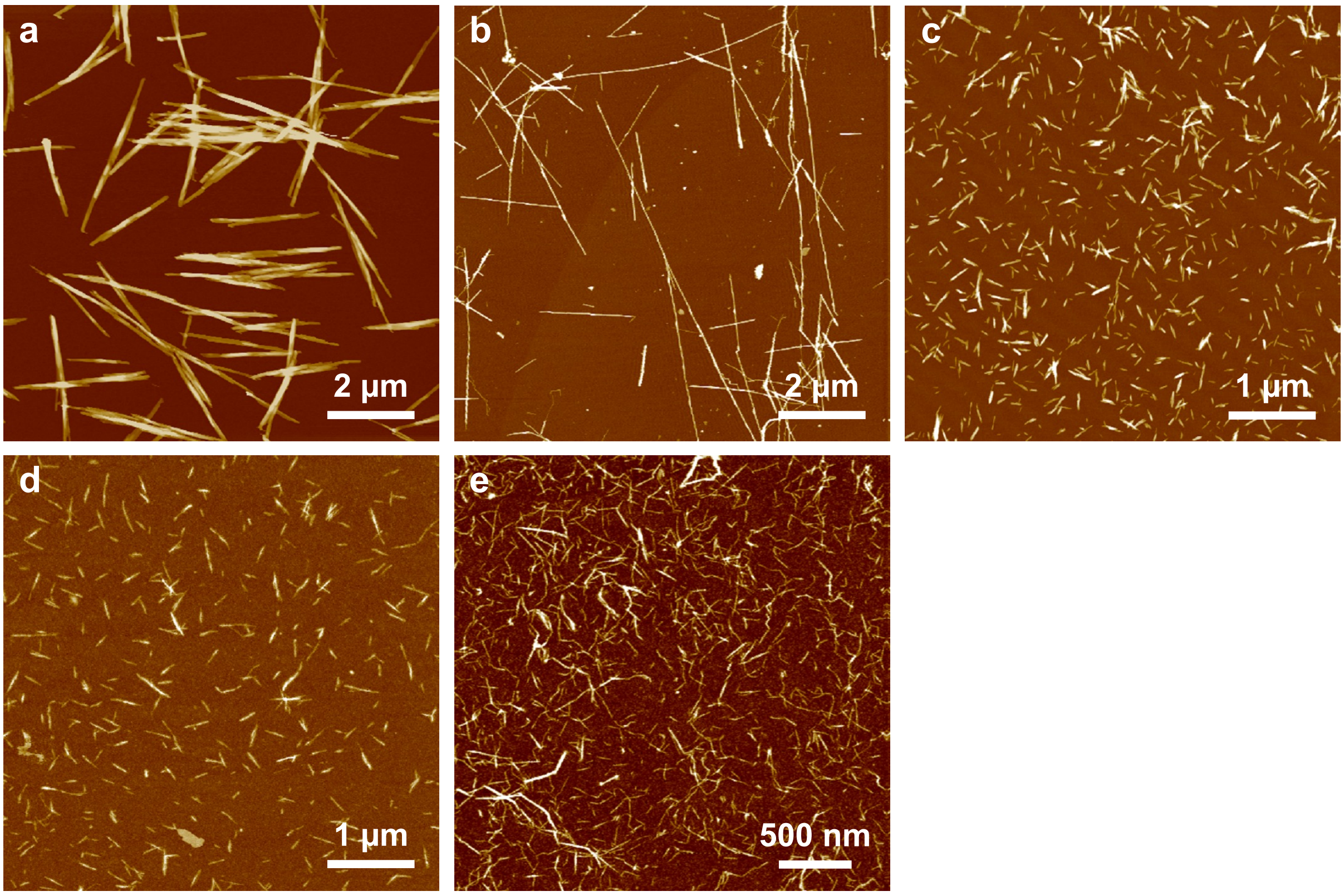

Figure S2. *Selection of AFM images used for the determination of nanocellulose dimensions (length, width, and height) among all images analyzed to obtain the values presented in* ***Table S1****. The AFM images show $tCNC_{CV}$ (a), $tCNC_{Lab}$ (b), cCNC (c), wCNC (d) and wCNF (e) submonolayers deposited by dipping. Image z-ranges are 60, 24, 30, 20 and 12 nm, respectively.*

The length and width of nanocelluloses were measured using ImageJ (National Institute of Health, U.S.A),[1] while the height was measured using the "section" tool in the Nanoscope software. Average values of length (l), width (w), and height (h) were obtained from at least 50 random measurements of each dimension for each type of nanocellulose. Due to the limited sharpness of the tips and their potential degradation over time, it is important to account for their impact on the measurements obtained from AFM images. In fact, the in-plane dimensions are typically overestimated due to the convolution between the tip shape and the object shape. To minimize this effect, a correction of 5 nm (corresponding to the average influence of the tip defects) was subtracted from the values obtained from Peakforce mode images and 10 nm for those obtained from Tapping mode images. The larger

tip size used in Tapping mode relative to Peakforce mode which explains the higher deconvolution needed for the tapping mode images.

Since all the nanocellulose samples used in this study did not possess square cross-sections, a 3D aspect ratio was determined using all three dimensions according to the equation S1 proposed by Parton et al.[2] (**Table S1**):

$$3D\ aspect\ ratio = \frac{l}{\sqrt{w*h}} \qquad \text{(S1)}$$

Table S1. *Dimensions of nanocelluloses determined by AFM from submonolayers deposited on PEI-coated silicon wafers by dipping and their corresponding mean 3D aspect ratio.*

Nanocellulose acronym	Origin/name (*supplier*)	Nanocellulose dimensions (nm)		Mean 3D aspect ratio
wCNC	Wood (*Cellulose Lab*)	Length	193 ± 79	≈ 14
		Width	39 ± 8	
		Height	5 ± 2	
cCNC	Cotton (*CERMAV*)	Length	202 ± 52	≈ 12
		Width	30 ± 12	
		Height	9 ± 5	
$tCNC_{Lab}$	Tunicate (*Cellulose Lab*)	Length	1,179 ± 1,005	≈ 90
		Width	20 ± 13	
		Height	9 ± 2	
$tCNC_{cv}$	Tunicate (*CERMAV*)	Length	2,228 ± 698	≈ 50
		Width	82 ± 32	
		Height	25 ± 9	
wCNF	Wood (*Cellulose Lab*)	Length	289 ± 124	≈ 40
		Width	17 ± 8	
		Height	3 ± 1	

2. Influence of the dipping time on film growth

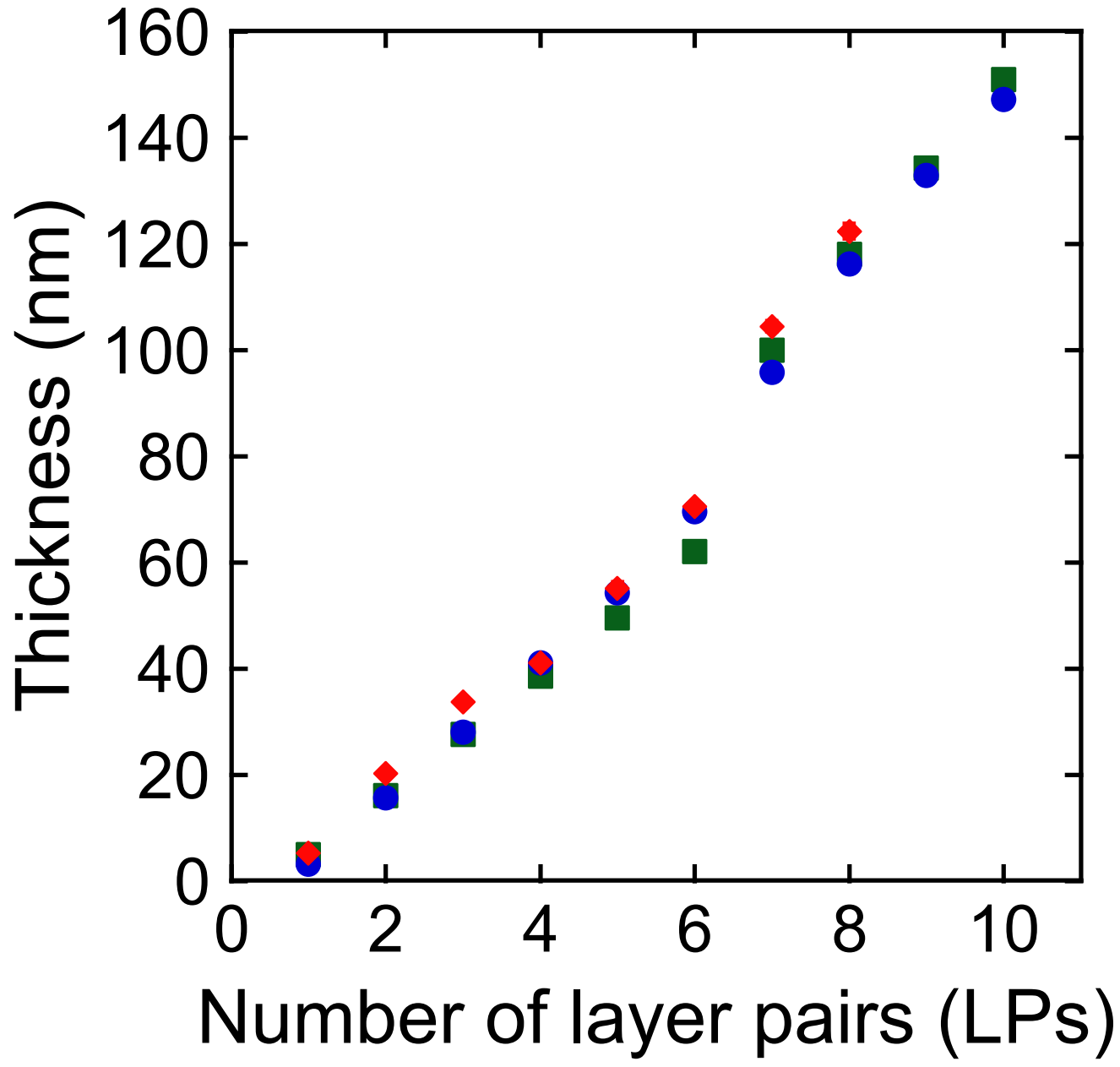

Figure S3. *Variation of the thickness of (tCNC$_{CV}$/PVAm)$_n$ films prepared with various dipping times (5 min (green), 10 min (blue), and 1 h (red)) as a function of the number of layer pairs.*

3. Influence of the nanocellulose nature on film growth

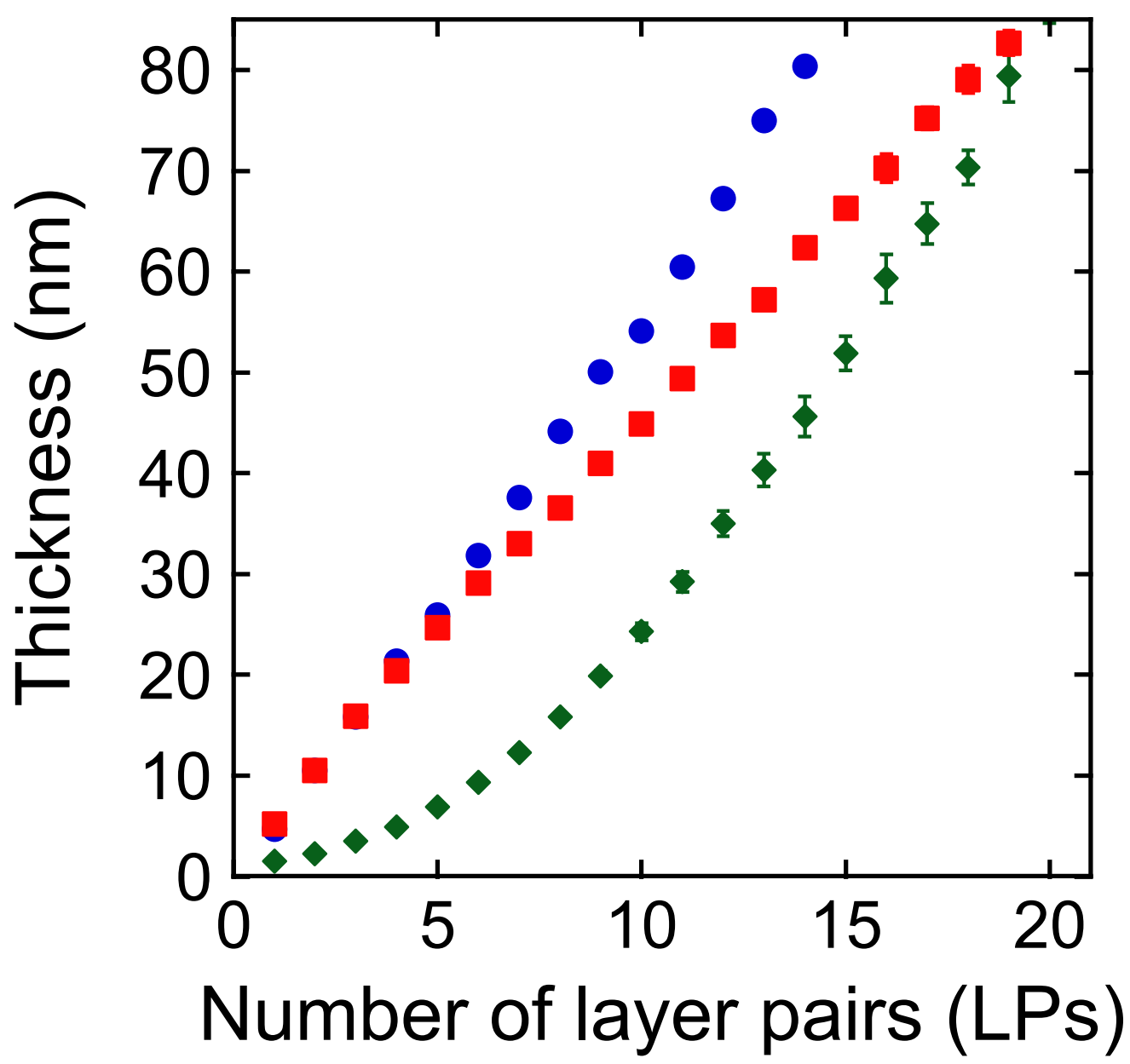

Figure S4. *Variation of the thickness (cCNC/PVAm)$_n$ (blue), (wCNC/PVAm)$_n$ (red), and (wCNF/PVAm)$_n$ (green) films built by dipping as a function of the number of layer pairs. Error bars are shown in each graph, but when they are indistinguishable, it is because they are smaller than the point size.*

4. Film thickness measurement using AFM

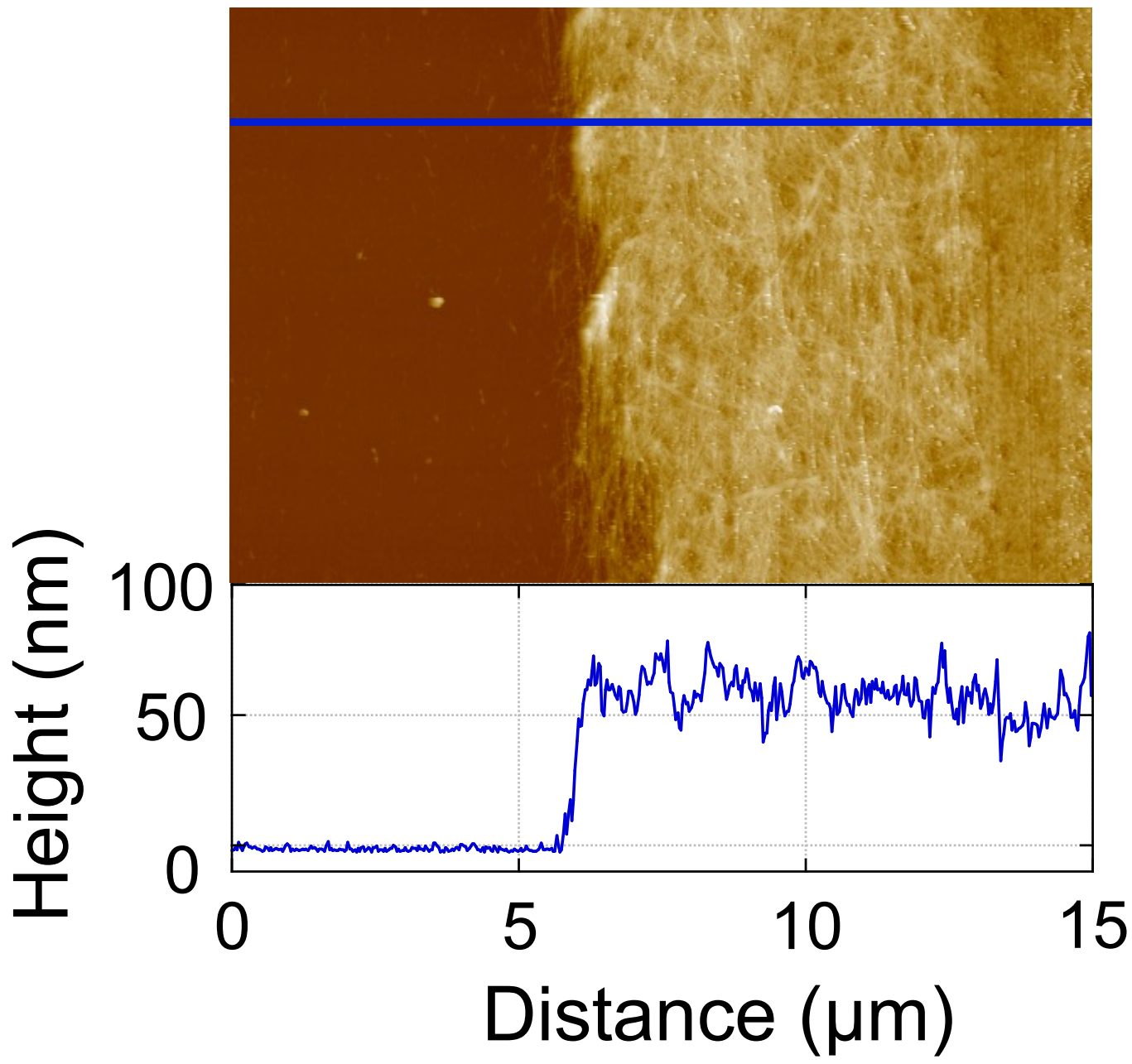

Figure S5. *(top) Topographical AFM image of the (tCNC$_{CV}$/PVAm)$_9$ film prepared by dipping and (bottom) the corresponding height profile.*

5. Ellipsometric spectra measured for different numbers of deposited layers

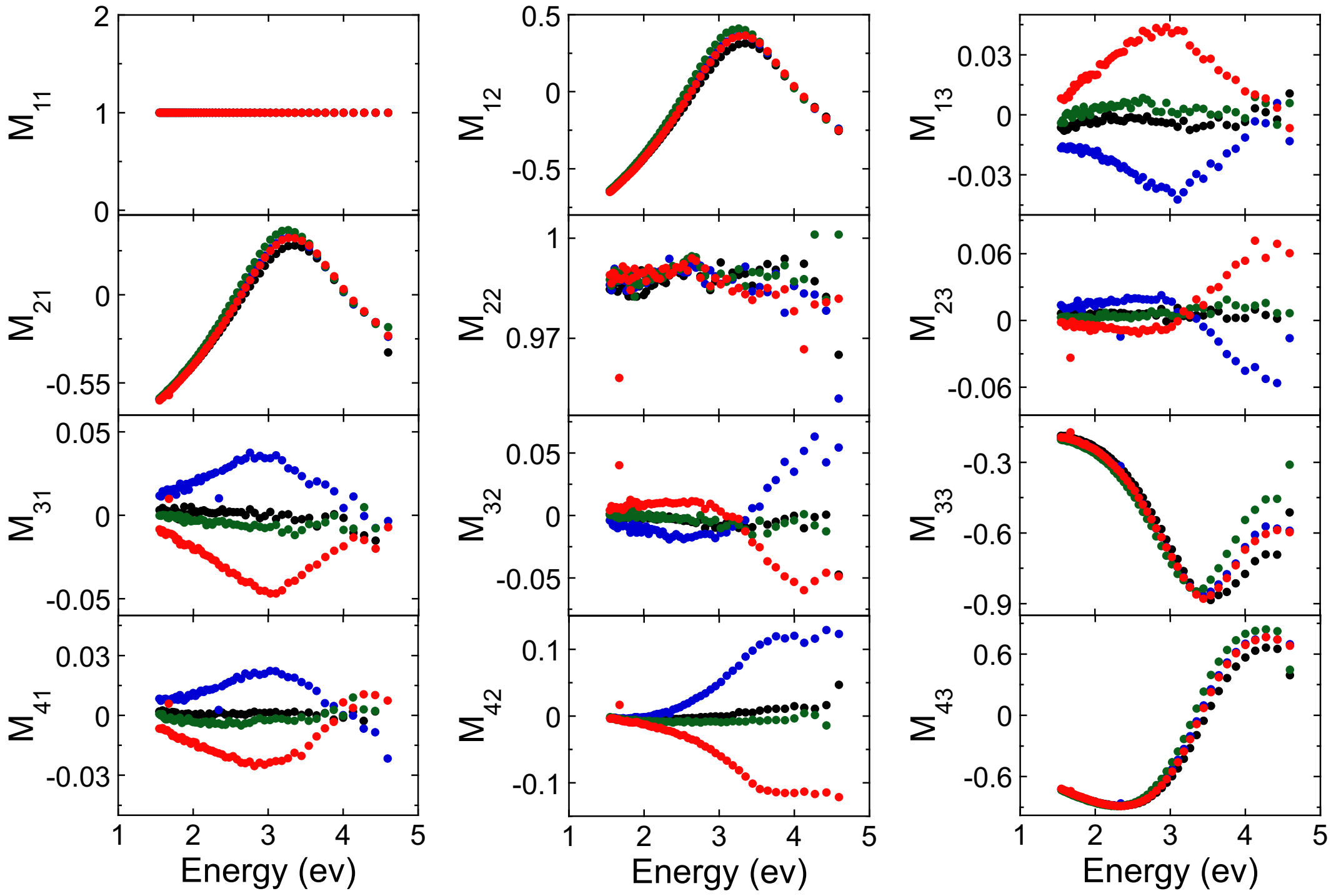

Figure S6. *Mueller matrix of ($tCNC_{Lab}$/PVAm)$_9$ measured at 4 azimuths (0 (black), 45 (blue), 90 (green) and -45° (red)).*

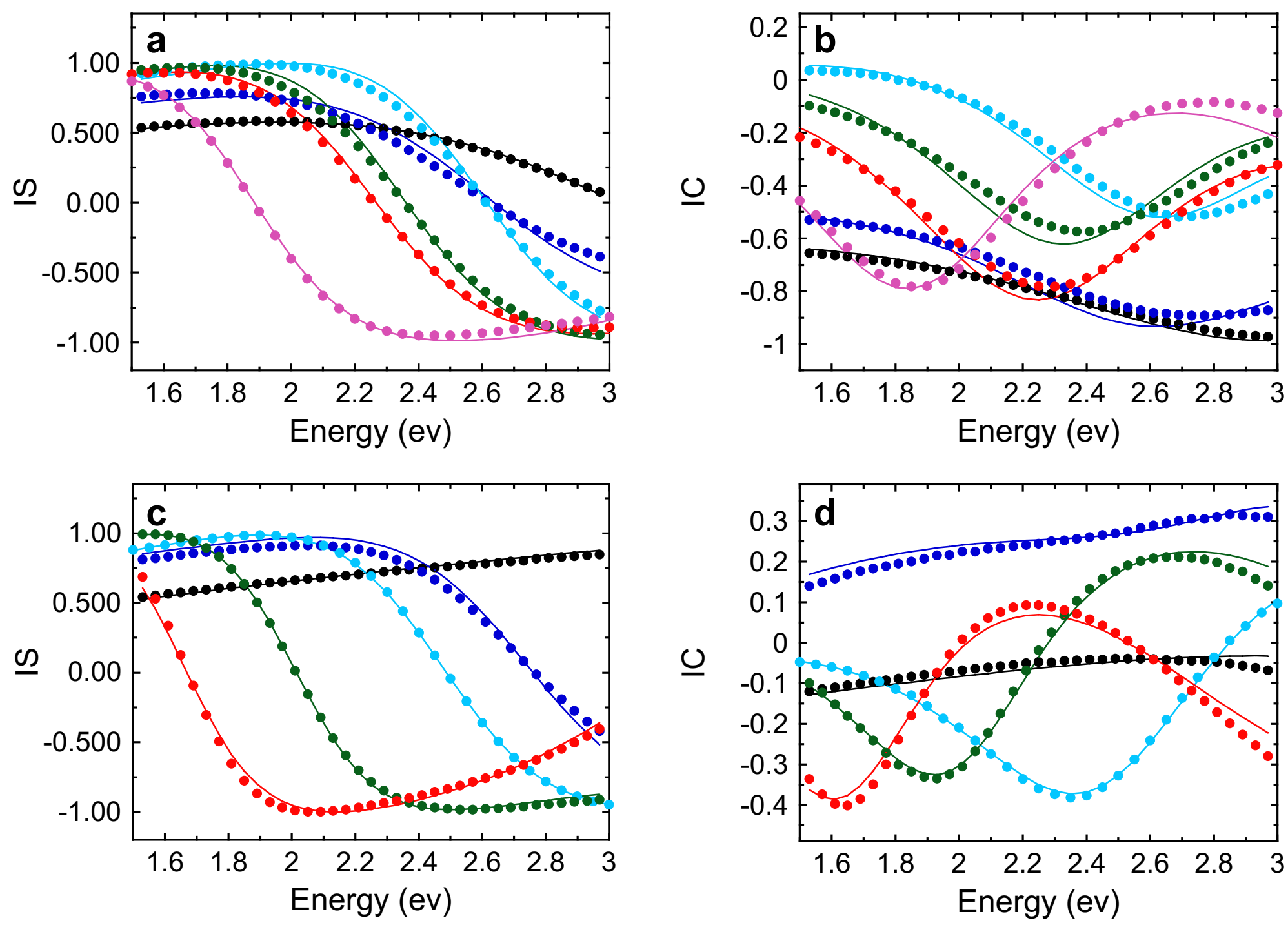

Figure S7. *Measured (dots) and modeled (solid lines) of Is (a,c) and) Ic (b,d) ellipsometric spectra of ($tCNC_{Lab}$/PVAm)$_n$ (a,b) (9 (black), 10 (dark blue), 11 (light blue), 12 (green), 13 (red) and 15 LPs (pink)) and ($tCNC_{CV}$/PVAm)$_n$ (c,d) (4 (black), 6 (dark blue), 8 (light blue), 9 (green) and 11 (red)) films. The azimuth was set to 0°.*

6. Influence of the substrate nature

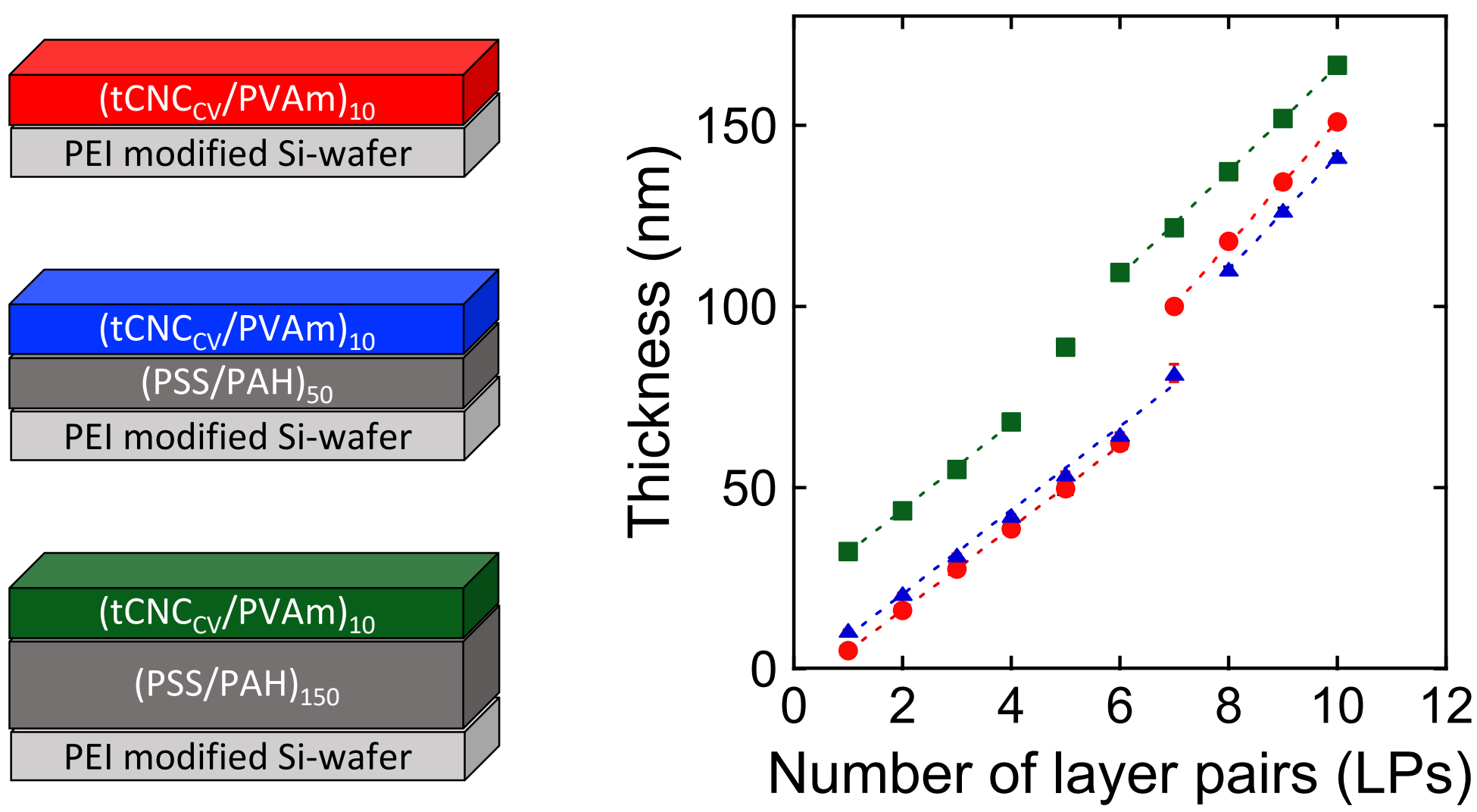

Figure S8. *(left) Schematic representation of (tCNC$_{CV}$/PVAm)$_{10}$, (PSS/PAH)$_{50}$(tCNC$_{CV}$/PVAm)$_{10}$, and (PSS/PAH)$_{150}$(tCNC$_{CV}$/PVAm)$_{10}$ films. (right) Variation of the thickness of (tCNC$_{CV}$/PVAm)$_{10}$ (red), (PSS/PAH)$_{50}$(tCNC$_{CV}$/PVAm)$_{10}$ (blue), and (PSS/PAH)$_{150}$(tCNC$_{CV}$/PVAm)$_{10}$ (green) multilayered nanocomposites as a function of the LP number of (tCNC$_{CV}$/PVAm)$_n$. Error bars are shown in each graph, but when they are indistinguishable, it is because they are smaller than the point size.*

7. Influence of the pH of the PVAm solution on film growth

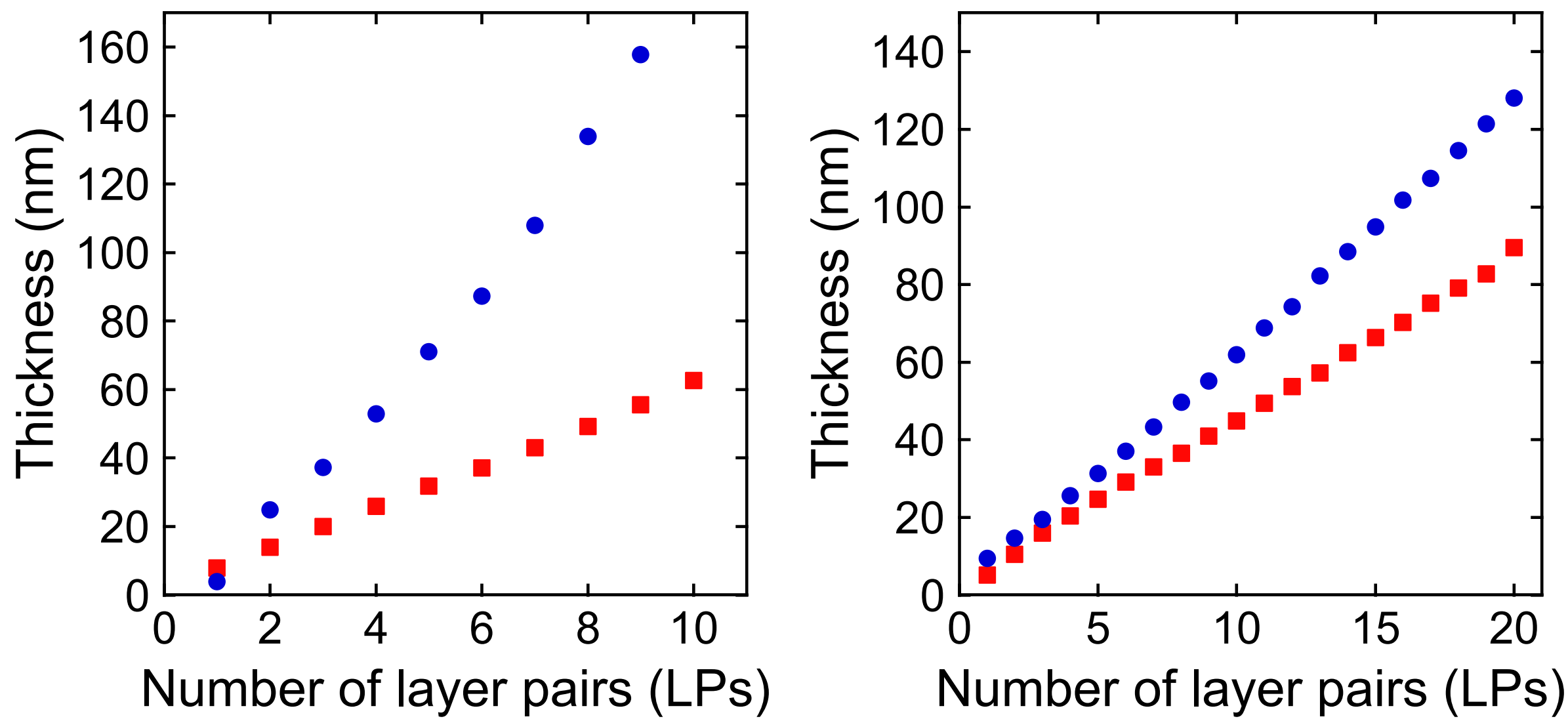

Figure S9. *Variation of the thickness of (cCNC/PVAm)$_n$ (left) and (wCNC/PVAm)$_n$ (right) films prepared with a PVAm solution at unmodified pH (around 6) (red) and modified pH (around 10) (blue) by dipping as a function of the number of layer pairs. Error bars are shown in each graph, but when they are indistinguishable, it is because they are smaller than the point size.*

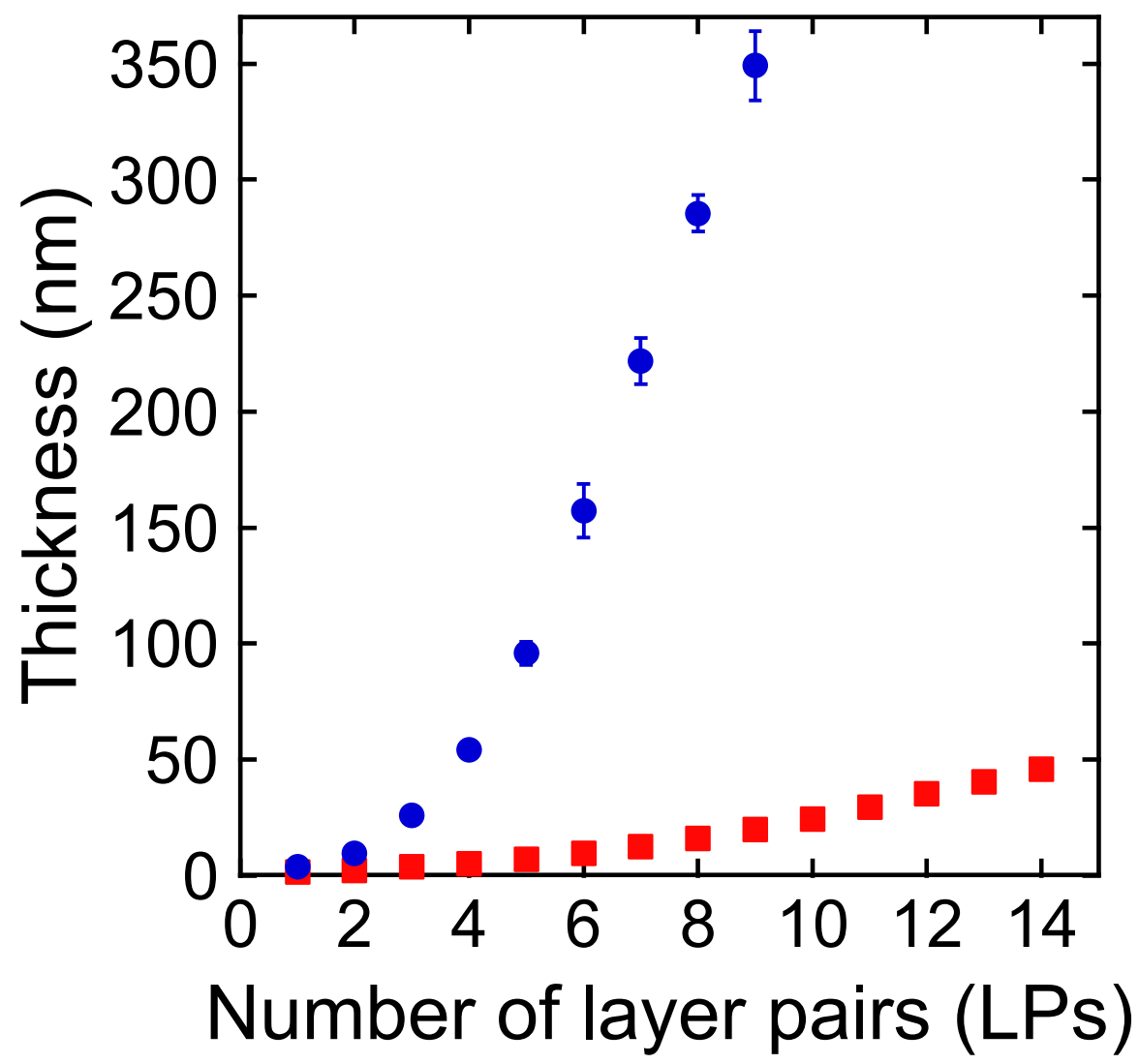

Figure S10. *Variation of the thickness of (wCNF/PVAm)$_n$ films prepared with a PVAm solution at unmodified pH (around 6) (red) and modified pH (around 10) (blue) by dipping as a function of the number of layer pairs. Error bars are shown in each graph, but when they are indistinguishable, it is because they are smaller than the point size.*

References

(1) Rezakhaniha, R.; Agianniotis, A.; Schrauwen, J. T. C.; Griffa, A.; Sage, D.; Bouten, C. V. C.; Van De Vosse, F. N.; Unser, M.; Stergiopulos, N. Experimental Investigation of Collagen Waviness and Orientation in the Arterial Adventitia using Confocal Laser Scanning Microscopy. *Biomech. Model. Mechanobiol.* **2012**, *11*, 461–473.

(2) Parton, T. G.; Parker, R. M.; van de Kerkhof, G. T.; Narkevicius, A.; Haataja, J. S.; Frka-Petesic, B.; Vignolini, S. Chiral Self-Assembly of Cellulose Nanocrystals is Driven by Crystallite Bundles. *Nat. Commun.* **2022**, *13*, No. 2657.
